# Bioinspired Morphing in Aerodynamics and Hydrodynamics: Engineering Innovations for Aerospace and Renewable Energy

**DOI:** 10.3390/biomimetics10070427

**Published:** 2025-07-01

**Authors:** Farzeen Shahid, Maqusud Alam, Jin-Young Park, Young Choi, Chan-Jeong Park, Hyung-Keun Park, Chang-Yong Yi

**Affiliations:** 1 Intelligent Construction Automation Centre, Kyungpook National University, 80 Daehak-ro, Buk-gu, Daegu 41566, Republic of Korea; farzeen.shahid@hotmail.com (F.S.); maqusud1@outlook.com (M.A.); jinypark@knu.ac.kr (J.-Y.P.); tpms1018@knu.ac.kr (C.-J.P.); parkhk@chungbuk.ac.kr (H.-K.P.); 2 Earth Turbine, 36, Dongdeok-ro 40-gil, Jung-gu, Daegu 41905, Republic of Korea; youngch5@naver.com

**Keywords:** bioinspiration, morphing, aerodynamics, hydrodynamics, renewables, energy, actuation, optimization

## Abstract

Bioinspired morphing offers a powerful route to higher aerodynamic and hydrodynamic efficiency. Birds reposition feathers, bats extend compliant membrane wings, and fish modulate fin stiffness, tailoring lift, drag, and thrust in real time. To capture these advantages, engineers are developing airfoils, rotor blades, and hydrofoils that actively change shape, reducing drag, improving maneuverability, and harvesting energy from unsteady flows. This review surveys over 296 studies, with primary emphasis on literature published between 2015 and 2025, distilling four biological archetypes—avian wing morphing, bat-wing elasticity, fish-fin compliance, and tubercled marine flippers—and tracing their translation into morphing aircraft, ornithopters, rotorcraft, unmanned aerial vehicles, and tidal or wave-energy converters. We compare experimental demonstrations and numerical simulations, identify consensus performance gains (up to 30% increase in lift-to-drag ratio, 4 dB noise reduction, and 15% boost in propulsive or power-capture efficiency), and analyze materials, actuation, control strategies, certification, and durability as the main barriers to deployment. Advances in multifunctional composites, electroactive polymers, and model-based adaptive control have moved prototypes from laboratory proof-of-concept toward field testing. Continued collaboration among biology, materials science, control engineering, and fluid dynamics is essential to unlock robust, scalable morphing technologies that meet future efficiency and sustainability targets.

## 1. Introduction

The modern era of engineering design is experiencing a growing convergence between biology and technology, driven by the goal of fabricating more efficient, adaptable, and sustainable systems. Research into bioinspired morphing for aerodynamics and hydrodynamics is garnering increasing attention because it provides solutions to key limitations of fluid dynamics, structural adaptability, and energy efficiency. By observing the evolution and capability of organisms, such as birds, bats, insects, and fish, to move through air and water with remarkable ability, engineers have discovered valuable insights that guide the design of shape-shifting or “morphing” structures and mechanisms. These adaptable systems have potential applications across various fields, including aerospace (adjustable aircraft components for drag reduction and lift enhancement) and renewable energy (transformable hydrofoils or wind turbine blades for maximizing energy extraction in fluctuating flow conditions).

Morphing refers to an entity’s ability to change its shape or structural configuration in response to external stimuli or internal demands. The importance of shape adaptability is evident in nature: birds vary their wing morphology to optimize soaring, maneuverability, or diving, and fish alter their fin angles and body curvature to balance propulsion efficiency and maneuverability. Nature’s versatile solutions have been refined over millions of years of evolution, offering what might be termed “ready-made” design templates. The study and translation of these designs into engineering applications can yield structural and functional improvements often unmatched by conventional, rigid designs.

The Introduction Section provides a comprehensive overview of the field of bioinspired morphing in fluid dynamics, focusing on the intersection of aerodynamics and hydrodynamics for aerospace and renewable energy systems. This review begins with a brief history of morphing concepts in engineering and biology, followed by an investigation of key bioinspired principles, the significance of morphing in fluid dynamics, and the major engineering domains that stand to benefit. Subsequently, we conclude by outlining the scope and structure of this review, emphasizing the need for interdisciplinary collaboration, encompassing biology, materials science, fluid dynamics, and robotics, to exploit nature’s morphing strategies comprehensively.

### 1.1. Historical Development of Morphing Concepts

The idea that nature can inspire human designs is not novel. Early developments in aeronautics heavily depended on observations of birds. The Wright brothers famously studied pigeon flight to glean insights into wing-warping, an early form of morphing technology [1]. Prior to that, Leonardo da Vinci’s sketches on birds and flying machines laid the foundation for bioinspired design centuries ago. In naval architecture, ancient civilizations observed the streamlined shapes of fish and marine mammals to develop more efficient hulls.

However, the term morphing in modern engineering contexts started emerging in the late 20th century, facilitated by advancements in smart materials (e.g., piezoelectrics, shape memory alloys (SMAs), magnetostrictive materials) that respond to external stimuli with controllable deformation [2]. Concurrently, improvements in computational tools for fluid and structural analysis have enabled engineers to model increasingly complex morphing configurations. By the late 1990s and early 2000s, research on morphing wing designs surged under programs sponsored by agencies like NASA and DARPA, focusing on seamless leading and trailing edges, flexible skins, and variable-camber airfoils [3].

In underwater robotics, bioinspired morphing garnered significant attention with the emergence of fish-like designs for autonomous underwater vehicles (AUVs). Drawing inspiration from the lateral undulation observed in fish and the oscillatory movements of rays, these engineered systems harness unsteady hydrodynamics to attain propulsion efficiencies that typically exceed those achievable with rigid propellers.

### 1.2. Key Bioinspirational Principles

Evolutionary Optimization in Nature: A compelling argument for studying biological systems is the premise that evolution has operated as a natural optimization process spanning millions of years. In air or aquatic environments, organisms that master shape adaptability gain substantial advantages in locomotion and survival. By analyzing features such as the drag-reducing feathers of birds, the turbulence-damping membranes of bats, or the dynamically controlled fins of fish, engineers can uncover specialized mechanisms for high-performance, shape-adaptive solutions [4].

Multidisciplinary Integration: Bioinspiration is inherently multidisciplinary, converging biology, materials science, mechanical engineering, aerospace engineering, robotics, fluid dynamics, and control systems. Collaboration between biologists and engineers is essential to decode nature’s complex systems, thereby uncovering novel insights into mechanisms such as the synergistic action of structural elasticity and distributed sensing for rapid, large-scale shape changes [5].

Challenges in Translating Biological Complexity: Although nature’s solutions can be remarkably effective, they are also highly integrated at multiple levels. Bird wings, for example, rely on interdependent bones, muscles, ligaments, and feathers, each with unique mechanical and sensory properties. Replicating such complexity directly is significantly challenging. Hence, to create more tractable morphing systems, engineers usually simplify nature’s designs, isolating certain functional aspects (e.g., flexible leading edges, variable camber) [6].

### 1.3. Significance of Morphing in Fluid Dynamics

#### 1.3.1. Importance of Shape Adaptability

In both aerodynamics and hydrodynamics, surface-fluid interactions are crucial for performance. Conventional systems often rely on fixed-geometry components optimized for a limited range of operating conditions. In contrast, morphing structures adapt continuously to maintain optimal flow characteristics across diverse regimes. Accordingly, aircraft can alter wing camber during take-off to generate lift and then streamline their wing profile for cruise flight to reduce drag; similarly, marine hydrofoils can modulate their curvature or angle to harness wave or tidal energy more effectively [7].

#### 1.3.2. Relationship Between Aerodynamics and Hydrodynamics

Although aerodynamics and hydrodynamics are often treated separately, they share foundational fluid motion equations. Design solution strategies in one domain can pave the way for insights in another. A bird-inspired wing could inspire a fish-inspired fin if both involve shape adaptability to control flow separation or maximize lift and thrust. In renewable energy, adaptive blades benefit both wind turbines (aerodynamic loading) and tidal turbines, reinforcing the commonalities between these fields [8].

### 1.4. Major Engineering Domains: Aeromechanics and Renewable Energy

#### 1.4.1. Aeromechanics Applications

In aerospace, morphing technology appears in applications ranging from full-scale adaptive aircraft wings to rotorcraft blades and small drones. Aircraft efficiency relies on minimizing weight and drag while maximizing lift. Morphing wings can adjust their aerodynamic profiles in flight, reducing fuel burn and potentially mitigating noise. Military and unmanned aerial vehicle (UAV) applications require different aerodynamic profiles for stealth, high-altitude cruising, or low-speed maneuvers, and morphing systems can seamlessly satisfy these requirements in real time. Early prototypes often relied on segmented or hinged sections; however, recent designs adopt continuous, flexible skins driven by smart materials such as SMAs or electroactive polymers (EAPs) [3].

#### 1.4.2. Renewable Energy Systems

The global demand for green energy has elevated interest in morphing concepts for wind and marine devices. Conventional wind turbines exhibit peak efficiency within a limited range of wind speeds; however, a morphing blade could dynamically alter its pitch or camber to optimize energy capture while mitigating stress from gusts. Similarly, marine energy devices operate in variable flow conditions, and bioinspired fins or flexible blades can adapt to changes in current or wave characteristics. Wave energy converters (WECs) that mimic flapping wing or fin motions can benefit further from shape-changing elements to match incoming wave profiles more precisely, enhancing overall performance and reliability [9].

### 1.5. Scope and Overview of the Review

This review examines how animals achieve—and engineers emulate—adaptive shape changes to boost aerodynamic and hydrodynamic performance. It first extracts the key geometric and material tricks used by birds, bats, insects, fish, and marine mammals (Section 2) and then explains the underlying unsteady fluid–structure physics that make those tricks effective (Section 3). Building on that foundation, Section 4 and Section 5 benchmark the latest morphing wings, blades, and fins, highlighting common gains in lift-to-drag, thrust, stall-resistance, and energy capture, while flagging domain-specific constraints such as compressibility in air and added mass in water. Section 6 surveys the experimental, computational, and data-driven tools that now let researchers probe and optimize these systems. Section 7 synthesizes the outstanding engineering hurdles—durability, actuation bandwidth, sensing-control integration, and certification—and sketches research priorities. Finally, Section 8 maps the broader impact across aviation, renewable energy, marine propulsion, and soft robotics, while Section 9 concludes with actionable directions for delivering robust, scalable morphing technologies.

## 2. Overview of Morphological Adaptation in Nature

Organisms interact with and modify their environments. Evolutionary selective pressures, including predation, resource competition, and environmental variability, drive the development of structures capable of responding to changing conditions over extended timescales. Biologically, morphing entails alterations in shape, stiffness, or surface characteristics that enhance flight control, swimming efficiency, or energy expenditure [10].

Morphing in species involves systems of bones or exoskeletons, muscles, connective tissues, and specialized sensory arrays such as mechanoreceptors, lateral lines, or hair-like receptors in feathers, fish, or bats, respectively. These systems work together to achieve continuous or discrete changes in form. Although traditional human designs have focused on rigid machines for predictability and simplicity, other biological systems often prioritize adaptability and multifunctionality—core themes informing modern morphing technology efforts [11].

### 2.1. Morphing Mechanisms in Natural Flyers

**Figure 1:**
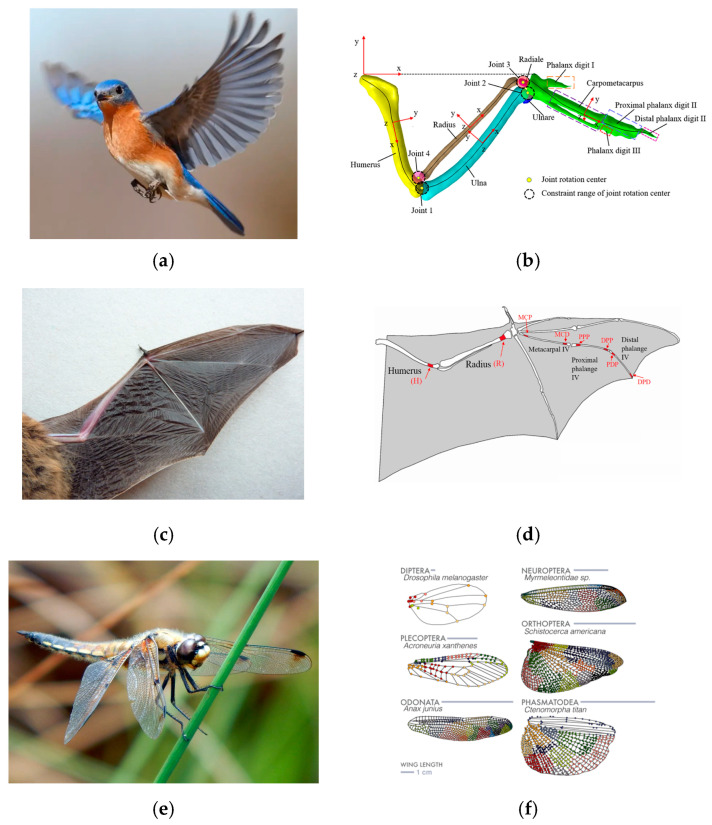
Wing anatomy of natural flyers (**a**) Bird flapping its wings. (**b**) Anatomical structure of a bird wing illustrating the arrangement of feathers (reproduced with permission from [12]). (**c**) Close-up view of a bat wing. (**d**) Bat wing illustrating the underlying skeletal framework and patagium (reproduced with permission from [13]). (**e**) Dragonfly displaying four distinct wings. (**f**) Insect wing morphology highlights its shape and venation patterns (reproduced with permission from [14]).

The skies host an incredible diversity of flyers, each employing specialized aerodynamic strategies to navigate complex environments. Although capable of powered flight, birds, bats, and insects have evolved distinct morphing mechanisms for lift generation, drag reduction, and maneuverability. These examples underscore the variety of ways flexible structures, muscle control, and refined aerodynamics can converge to produce remarkable flight performance. Figure 1 presents the wing anatomy of natural flyers, illustrating the wing structures and features of birds [12], bats [13], and insects [14].

#### 2.1.1. Birds

##### Diversity of Avian Wing Morphology

**Figure 2:**
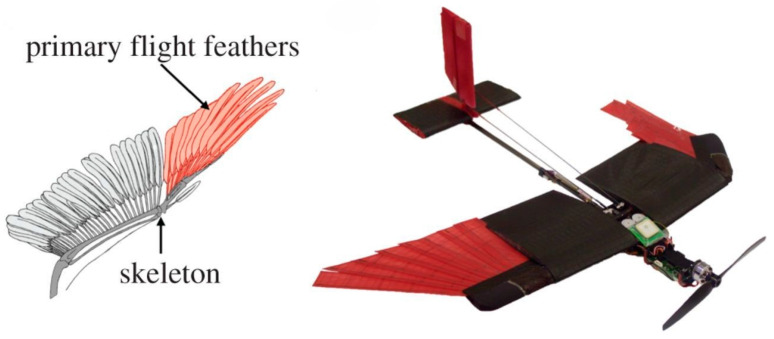
Anatomy of a bird’s wing alongside a morphing wing drone (Reproduce with permission from [17]).

Birds represent one of the most extensively studied groups regarding morphing flight structures. The avian wing is an intricately integrated system comprising bones (humerus, radius, ulna, carpometacarpus, phalanges), flight muscles, and a complex arrangement of feathers [15,16]. Figure 2 (ref. [17]) presents a schematic of a bird’s wing anatomy, highlighting an articulated skeleton and overlapping flight feathers. It illustrates how birds reduce their wing area by folding their outer primary feathers against the skeleton, a natural morphing strategy for high-speed flight. This skeletal–muscular–feather complex allows birds to modulate their wing shape and area, adapt their wingspan, and even alter the surface microstructures of their feathers. Soaring birds such as albatrosses minimize wing loading by extending their long wings, whereas hawks retract and sweep their wings during high-speed dives [18].

##### Wing Flexion and Extension

Flexion and extension at the shoulder, elbow, and wrist joints, primary morphing actions, enable birds to fold their wings tightly or extend them broadly [19].

Nature-to-Device Bridge: *Avian joint flexion guides Lis-Eagle morphing joints, enhancing roll maneuverability.*

**Figure 3:**
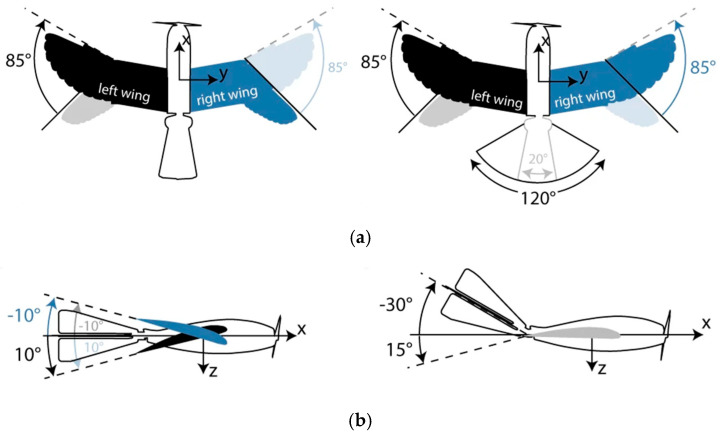
Flight configurations and control mechanisms of the Lis-Eagle drone (**a**) Asymmetric wing folding and pitching; (**b**) Lift augmentation during banked maneuvers accomplished by extending wing and tail surfaces to increase area and upward tail deflection (reproduced with permission from [21]).

Moreover, certain species like owls morph their leading-edge feathers to damp turbulence and achieve silent flight. These structural modifications illustrate how subtle adjustments minimize noise or turbulence in natural flight [20]. Figure 3 (ref. [21]) illustrates the Lis-Eagle drone’s morphing configurations and aerodynamic control strategies, including roll moment generation via asymmetric wing folding or pitching (Figure 3a), including lift augmentation via the wing/tail extension and tail deflection, demonstrating its adaptive design for enhanced flight efficiency and maneuverability (Figure 3b).

Benchmark comparison: Building on avian wing-morphing principles, the Lis-Eagle drone integrates SMA-based twist actuators within a carbon–flexure skin to vary camber ± 8° and spanwise twist ± 6° in real time. Wind-tunnel measurements show a 12% lift-to-drag increase in cruise and 25% higher gust-load attenuation relative to a rigid-wing tilt-rotor of equal mass, though hover endurance falls 22% because of actuator power draw. In confined-space tests, the Lis-Eagle negotiates turning radii 35% tighter than a DJI-M300-class quad-rotor, yet its payload fraction drops from 24% to 18% owing to morphing-mechanism mass. These quantified trade-offs illustrate both the aerodynamic promise and current structural-integration challenges of bio-inspired morphing.

##### Feather Articulation and Slotting

Feathers can autonomously articulate and change orientation. For instance, primary feathers at the wingtips often separate into “finger-like” slots at high angles of attack, mitigating drag and delaying stall. This phenomenon, termed “feather slotting”, effectively minimizes wingtip vortices. Birds like eagles and hawks exploit this mechanism to maintain lift during steep glides or low-speed maneuvering [22].

##### Variable Camber and Swept Wings

Swifts alter their wing sweep to adapt to different flight modes. When cruising at high speeds, they sweep their wings backward to minimize drag, like the concept employed in variable-sweep wing aircraft (e.g., certain military jets). When maneuvering, they spread their wings forward to increase lift and agility. The ability to change their camber (the curvature of the wing’s cross-sectional profile) is central to controlling lift and drag. Other birds adjust their camber by altering feather positions or wing-joint angles, fine-tuning aerodynamic performance over wide speed ranges. This dynamic camber control underlies much of the bird’s ability to optimize lift and drag spontaneously [23].

##### Aerodynamic Efficiency and Energy Conservation

Long-distance migratory birds like albatrosses exploit dynamic soaring by adjusting wing orientation to extract energy from wind gradients. Such strategies, combined with efficient wing morphing, can significantly conserve their energy over vast distances. The interaction between morphology and behavior highlights how subtle wing morphing enhances flight endurance and range [24].

#### 2.1.2. Bats

##### Membranous Wing Structure

Bats, the only flying mammals, possess wings formed by a thin elastic membrane stretched over elongated finger bones, unlike birds. This highly elastic membrane contains muscles, blood vessels, and sensory receptors that enable significant deformations. By activating small intrinsic muscles, bats adjust their local membrane tension, enabling fine control over wing curvature and twist [25].

##### High Maneuverability and Agile Flight

Bats excel at rapid turns, hovering, and landing upside-down. Their ability to adjust wing segments locally contributes significantly to their agility. In cluttered environments (e.g., dense forests), some species use high-lift, high-drag wing configurations to hover or pivot sharply during foraging. In contrast, others extend and stiffen their membranes for faster cruising. These styles often correlate with morphological and mechanical properties, such as wing aspect ratio, membrane thickness, and the arrangement of supporting bones, which highlight the adaptability of bat wings [26].

##### Distributed Sensing and Control

**Figure 4:**
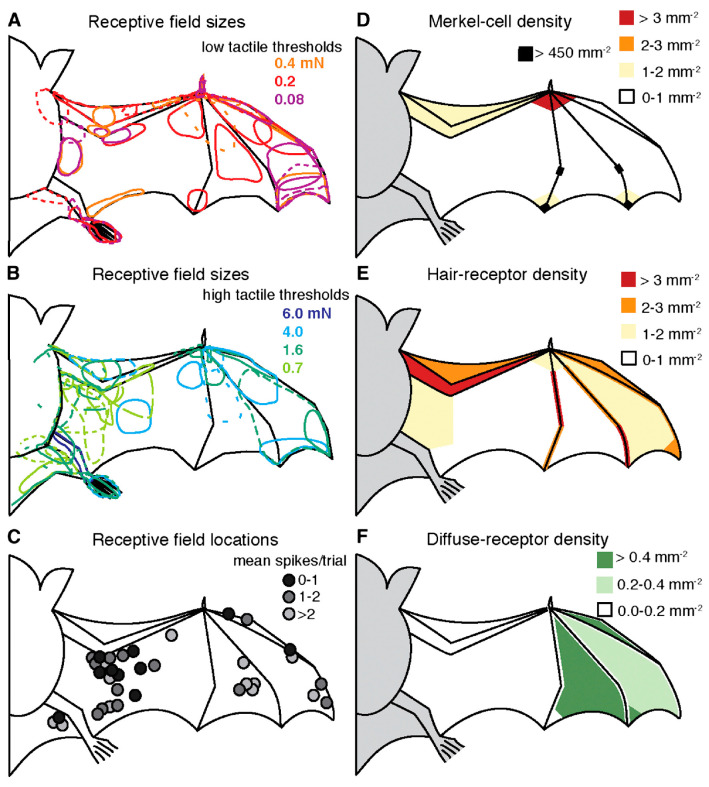
The receptive field characteristics and tactile sensitivity of somatosensory neurons, with color gradients indicating response thresholds determined by von Frey filament stimulation. (**A**) At the lowest von Frey loads (0.08–0.4 mN), the smallest and most sensitive receptive fields trace the leading edge and distal digits; (**B**) With stiffer filaments (0.7–6 mN) these fields expand and migrate toward the wing root, indicating higher force thresholds centrally; (**C**) Circles mark regions activated by a calibrated air jet; darker and larger symbols denote higher spike rates, concentrating flow sensitivity near the dactylopatagium and arm–wing junction; (**D**) Merkel-cell clusters, shaded from pale to dark brown, reach their greatest density at the wingtip and leading margin; (**E**) Hair-type mechanoreceptors, shown in yellow–red tones, are similarly enriched along distal phalanges and the front edge; (**F**) Diffuse or unclassified receptors blanket the membrane at moderate density (green), ensuring continuous proprioceptive coverage. Together, these maps link tactile thresholds, airflow responsiveness, and receptor distribution to illustrate the wing’s finely tuned somatosensory design(Reproduce with permission from [28]).

Bat wings feature minute sensory hairs and Merkel cell complexes providing tactile feedback on airflow and wing deformation. The bat wing contains a distinctive array of sensory neurons specialized for detecting airflow and tactile stimuli. These sensory signals play a crucial role in informing flight behavior, yet the neural pathways involved in wing sensorimotor processing remain largely unexplored (results presented in Figure 4) [27].

##### Potential for Bioinspired Materials

Bat wing membranes contain elastin and collagen, combining the benefits of flexibility and strength [29]. Previous research [30] investigated the role of elastin fibers in the mechanical behavior of bat wing membranes, emphasizing their contribution to the wing’s extensibility and structural integrity. Although bat wing membranes and stretchable, self-healing aircraft skins remain insufficiently studied, biomaterials and bioinspired engineering research indicate possible future advancements in this field.

#### 2.1.3. Insects

##### Exoskeleton-Based Wing Mechanisms

Insect wings typically attach to the thorax via specialized exoskeletal articulations, featuring thin membranes supported by a network of veins [31]. Rapid flapping (tens to hundreds of beats per second) combines wing rotation, twisting, and camber alterations to generate lift [32]. Despite their seemingly rigid appearance, insect wings undergo subtle yet basic deformations [33].

##### Wing Deformation and Leading-Edge Vortices (LEVs)

A key aerodynamic mechanism is the generation of LEVs during the downstroke, enhancing lift and delaying stall. Hawkmoths, fruit flies, and bees exploit stable LEVs to maneuver in highly unsteady flow environments [34,35]. Engineers mimic these unsteady aerodynamic properties in flapping-wing micro air vehicles (MAVs).

##### Resonant Flight and Power Efficiency

Insects (e.g., bees) utilize asynchronous flight muscles operating near the resonant frequency of the wing–thorax system, minimizing the energy expended while flapping [36]. Passive wing deformation further refines lift generation without requiring excessive muscular effort. This resonance-inspired approach guides MAV designs that require high efficiency from limited onboard power [37].

##### Deployable and Foldable Wings

Insects fold their wings away when inactive (e.g., beetles tucking hindwings beneath hardened elytra). Although primarily adaptations for defense and mobility, deployable wing mechanisms inform compact UAV designs where morphing wings can be stowed for storage and unfolded for flight [38].

### 2.2. Morphing Mechanisms in Natural Swimmers

A comparable array of morphing strategies can be observed below the water surface. Aquatic organisms, from the flexible undulation of eels and the dynamic flipper motions of whales to the jet propulsion of cephalopods, exemplify how shape adaptability confers advantages in propulsion and maneuvering.

#### 2.2.1. Fish

##### General Fish Locomotion

Fish employ diverse locomotor modes—ranging from anguilliform (full-body undulation) to carangiform and thunniform (primarily tail-driven propulsion)—each reflecting varying degrees of body flexibility and active morphing. Flexible body segments and fin rays enable fish to alter their curvature and stiffness distribution along the body, optimizing thrust and maneuverability [39]. Such adaptability emerges from active muscle contractions and passive mechanical properties, allowing fish to exploit fluid dynamic cues and enhance their swimming efficiency.

##### Fin Rays and Mechanically Tunable Fins

In teleost fish, fish rays are segmented bones connected by collagen or elastin-rich tissues, enabling bending and shape adaptation. By adjusting muscle tension on these rays, fish modulate fin stiffness in real time: high stiffness for rapid acceleration and lower stiffness for precise maneuvering or station-holding [39,40]. Advanced biomimetic robots designed for more efficient propulsion rely on this distributed control of stiffness as a key underlying mechanism, as already demonstrated by SMA-tendon fish that switch burst/cruise modes [41], cable-driven soft fins that tighten for agile turns [42], and self-learning composite rays that vary stiffness to maximize thrust [43].

##### Passive Flex Fins

The passive flexibility of fish fins can reduce drag and streamline flow interactions, while active morphing (via muscle activation) enhances maneuverability during abrupt turns or predator-prey interactions. Researchers have found that careful tuning of passive flexibility in robotic fins yields robust performance across a range of flow conditions without continuous high-energy actuation [44].

##### Specialized Morphing Examples: Flying Fish and Others

Flying fish can glide above the water surface by expanding their pectoral fins like wings. These fins morph into rigid airfoils, enabling extended glides to evade predators. This dual-purpose design, fins that function effectively in both water and air, demonstrates the versatility of their morphological adaptation [45].

#### 2.2.2. Cetaceans (Whales, Dolphins)

##### Flukes and Flippers

Cetaceans (e.g., dolphins, whales) generate thrust predominantly via vertical fluke oscillations, exploiting the dense medium of water to achieve significant accelerations and stable cruising [46]. Fluke edges often exhibit chordwise flexibility, maintaining favorable angles of attack and reducing flow separation [47]. Meanwhile, pectoral flippers, particularly in species like humpback whales, morph their leading edges for agile turns and improved stall characteristics [48].

##### Tubercles and Leading-Edge Control

Humpback whale flippers feature tubercles along the leading edge, which passively energize the boundary layer, effectively delaying stall [48]. Engineers have replicated these tubercles on wind turbine blades and underwater foils with notable improvements in lift performance and flow control. Although tubercles do not actively “move,” some cetaceans can subtly alter flipper orientation or local tissue tension to adjust flow interactions [49].

##### Bioinspired Marine Propulsion

Dolphin-inspired fluke designs attempt to mitigate cavitation and maintain efficiency over diverse speeds, incorporating flexible materials that can adapt shape under varying load conditions [47]. These bioinspired propulsors are often quieter, more maneuverable, and potentially more energy-efficient than conventional ship propellers, garnering significant interest in biomimetic underwater vehicle design. Laboratory and field studies report propulsive efficiencies of 75–92% for oscillating foils and cetacean-like flukes, typically 10–20 percentage points higher than equivalently loaded marine propellers [50,51].

#### 2.2.3. Cephalopods (Squids, Octopuses, and Cuttlefish)

##### Jet Propulsion and Body Deformation

**Figure 5:**
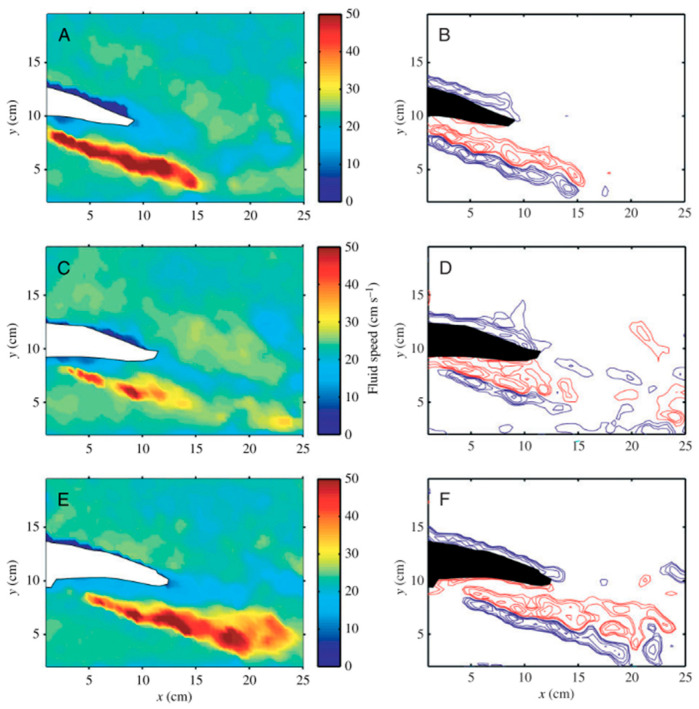
Colour maps of velocity (**A**,**C**,**E**) and their paired vorticity fields (**B**,**D**,**F**) capture flow generated by three individual jet pulses from an adult long-fin squid (Loligo pealei) maintaining position in a 25 cm s^−1^ flume (water moves left → right). In the vorticity panels (**B**,**D**,**F**), warm reds indicate positive (counter-clockwise) span-wise rotation, cool blues mark negative (clockwise) rotation, and deeper hues denote stronger vorticity. In velocity plots, the squid’s posterior body is masked in white; in vorticity plots, it appears black. The narrow, high-speed streak corresponds to the jet plume. Vorticity contour levels (rad s^−1^) are: (**B**) 4, 6, 10, 15, 20; (**D**) 2, 4, 6, 10, 15, 20; (**F**) 3, 4, 6, 10, 15, 20 (colour scale defined in Figure 2). Each panel pair is a snapshot taken at a characteristic instant within the jet cycle, not consecutive PIV frames: (**A**,**B**) t = 0.3 s, t/Δt^−^ = 1.0; (**C**,**D**) t = 0.7 s, t/Δt^−^ = 1.2; (**E**,**F**) t = 0.5 s, t/Δt^−^ = 0.9. Together, the images demonstrate how rhythmic mantle expansion and contraction redirect momentum, exemplifying the mantle-morphing strategy discussed in the manuscript text (reproduced with permission from [53]).

Cephalopods rely significantly on jet propulsion: the mantle cavity fills with water, which is then forcefully expelled through a siphon [52]. Several species also display mantle morphing, altering their volume and shape to adjust buoyancy, directional control, or body streamlining (presented by Figure 5) [53]. Octopuses and cuttlefish can compress their bodies to navigate tight spaces; a morphological flexibility rarely observed in rigid-bodied natural swimmers [54].

##### Fins and Undulating Membranes

In addition to jetting, squids and cuttlefish possess lateral fins that undulate for fine maneuverability. These fins may locally stiffen or relax via internal muscle control, allowing these cephalopods to hover, reverse, or achieve slow, precise movements [53]. Integrating such active fin morphing into soft robotic platforms has yielded designs capable of complex station-keeping in turbulent-flow environments [55].

##### Soft Robotics Inspiration

The near-complete absence of rigid skeletal elements in cephalopods provides a compelling model for soft robotics. Consequently, multiple research groups have developed entirely soft underwater robots capable of shape reconfiguration, mimicking the cephalopod’s distributed muscle architecture [56]. This morphing capability fosters robust adaptation to unstructured marine environments—an appealing trait for deep-sea exploration and delicate sampling tasks [57]. These advancements highlight the potential of cephalopod-inspired designs to overcome limitations of traditional rigid robotic systems in complex underwater environments.

### 2.3. Bioinspired Concept

**Figure 6:**
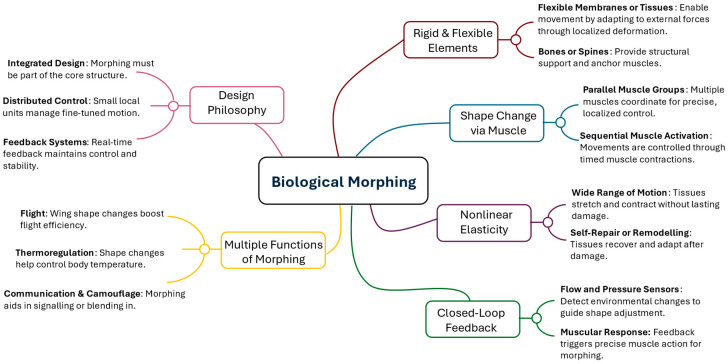
Concept map: Principles of biological morphing.

Bioinspiration encompasses two primary forms: (1) replicating natural motion patterns (e.g., flapping wings or undulating bodies), and (2) replicating the structural features (e.g., flexible membranes, specialized feathers, or fin arrangements) that enable these motions. These approaches are often integrated to mirror the kinematics of natural locomotion and emulate analogous material and structural arrangements. Figure 6 presents a concept map of Biological Morphing. Engineers seldom achieve exact replication of natural systems. Instead, they modify biological strategies to address design goals, material limitations, and operational conditions. Biological morphing solutions, refined through millions of years of evolutionary processes, are typically hierarchical, with macro-scale skeletal elements complemented by micro-scale features (such as muscle fibers or collagen layers), each contributing to overall adaptability.

Emerging Biological Inspirations: Although birds, bats, insects, fish, and cetaceans are frequently studied, other underexplored organisms also offer intriguing lessons in morphing. Shape adaptability for traversing mixed terrains (e.g., land vs. water) is demonstrated by deployable membranes in some reptiles and amphibians. Moreover, the capacity for morphing in coloration or texture, observed in certain marine and terrestrial species (e.g., cephalopods, chameleons), suggests the potential for multifunctional surfaces that could offer camouflage, flow control, or distributed sensing. Translating biological morphing into engineering contexts entails inspiration and abstraction. Engineers are required to distill essential functional principles (e.g., distributed actuation, embedded sensing, and passive elasticity) from complex living systems. Emerging approaches combine advanced composites or smart materials with bioinspired structural layouts to replicate the synergy of mechanical strength, elasticity, and feedback control. Bio-inspired concepts harness natural strategies for better performance, including improved aerodynamic efficiency, hydrodynamic stability, and adaptability. By synthesizing biological insights with modern engineering tools, researchers can accelerate innovation across sectors, from aerospace and automotive to soft robotics and renewable energy.

## 3. Physics of Bioinspired Morphing Systems and Mechanisms

### 3.1. Fundamental Principles of Morphing in Biological Systems

Bio-inspired morphing systems derive inspiration from a broad spectrum of natural adaptations.

Nature-to-Device Bridge: *Bat-wing membrane elasticity enables compliant UAV skins, improving low-speed agility.*

Nature-to-Device Bridge: *Fish-fin muscle actuation drives segmented hydrofoils, achieving high-amplitude camber modulation.*

These strategies are often seamlessly integrated into an organism’s skeletal and muscular architecture, optimizing locomotion efficiency and maneuverability [58,59]. In biology, morphing can be either intrinsically passive, where structural or material properties automatically adapt under varying load conditions (e.g., feather reorientation during gusts), or actively driven by muscle activity (e.g., bat wings and fish fin rays) [18,47]. Organisms frequently integrate both techniques, relying on passive elasticity to conserve energy while using active inputs for fine control [60,61].

#### 3.1.1. Wing Flexion and Extension in Birds

Such structural modifications provide a pathway for bioinspired engineering solutions to achieve noise reduction, a critical factor in both aviation and wind energy industries. Similar engineering parallels emerge in flapping-wing MAVs that replicate insect wing-vein structures, enabling dynamic twisting and bending essential for flight control [62].

#### 3.1.2. Passive vs. Active Morphing in Nature

In biology, passive morphing typically involves structures that bend, twist, or fold in response to external forces such as fluid pressure or gravity, without requiring active muscular effort. Active morphing is controlled by neuromuscular systems.

*Passive Morphing: Passive morphing relies on inherent material compliance. Birds’ covert feathers, for instance, partly self-adjust in response to airflow changes, reducing separation [63]. Fish fins also bend elastically under fluid forces, maintaining stable thrust across varying swimming speeds [47,64].*

*Active Morphing: Active morphing involves direct muscular or neural control over shape changes. Bats adjust local membrane tension, and insects rapidly twist wing sections for steering. Although more energy-intensive, active morphing provides fine-tuned control over flight or swimming dynamics, often critical for evading predators or complex maneuvers [18,60,65].*

Engineers have garnered insights from both passive and active morphing techniques. Flexible flapping MAVs emulate active insect or bat flight, while passively morphing trailing edges in commercial aircraft wings or wind turbine blades mimic how bird feathers reduce drag under varying flow conditions.

#### 3.1.3. Material and Structural Adaptations Enabling Morphing

Biological systems often feature composite-like tissues that combine stiff and flexible regions, allowing localized deformations without compromising overall integrity. Flexible membranes (in bats) or segmented bony rays (in fish fins) combined with elastic connectors provide robust scaffolding [59]. The leading edges of birds’ wings are stiff to maintain their aerodynamic shape, while the trailing edges are more flexible [63]. Bats employ elastic membranes embedded with muscle fibers, and insects use vein-reinforced membranes that twist in flapping flight [18,58]. Replicating these hierarchical composites in engineered morphing structures typically involves smart materials (e.g., SMAs, EAPs) embedded in flexible skins or segmented frames [66]. Distributing actuation points across the surface addresses the distributed muscle architecture, enabling localized shape control [67].

An example is bat-wing-inspired membranes:

*Engineers attempt to mimic these properties using advanced polymers and composites that can stretch, self-repair, and maintain aerodynamics under varying load conditions [68]. Shape memory polymers, for instance, can partially mimic the elastic deformation of bat skin [60]. Although conventional materials do not perfectly match the complexity of biological tissues, ongoing research in biomaterials, including genetically engineered or biohybrid materials, hints at future breakthroughs that may bridge this gap [65].*

Distributed sensors further approach the constructive interaction found in bats’ wings:

*While the remarkable adaptability of cephalopods is underpinned by a sophisticated, distributed nervous system that governs local muscle actuation [69], current cephalopod-inspired robots mainly replicate the soft, deformable body and distributed actuation, not the neural control itself [70]. Developing truly decentralized control that emulates biological neural networks remains an open challenge. Present systems, therefore, rely on centralized controllers or pre-programmed responses. Ongoing work explores embedded sensor–actuator arrays and machine-learning policies [42] to achieve more autonomous, adaptive behaviour, edging soft robots closer to their biological counterparts.*

#### 3.1.4. Energy Efficiency in Biological Morphing

Nature’s solutions to morphing problems tend to be highly energy efficient. Birds store and release elastic energy in wing tendons, fish utilize body undulations that resonate with fluid vortices, and insects exploit asynchronous flight muscles near the resonant frequency of the wing-thorax system [20].

*A variety of organisms balance the energetic demands of morphological changes by storing and releasing elastic energy within tendons, membranes, or fin rays. Birds exploit tendon elasticity during flapping cycles, and fish leverage body resonances for propulsion [18]. Such cyclic energy exchange reduces the net metabolic cost. In contrast, engineered systems often suffer from actuation inefficiencies unless carefully designed. Bioinspired approaches thus incorporate resonance-based flapping, compliant joints, or tuned stiffness to minimize power consumption during shape changes [71,72].*

### 3.2. FSIs in Morphing Systems

In bioinspired morphing, FSI drives performance: shape changes alter flow, which, in turn, modifies structural loads.

Nature-to-Device Bridge: *Fish-fin feedback inspires compliant hydrofoils with sensing, enabling adaptive FSI.*

Advanced computational fluid dynamics (CFD) tools enable engineers to model these coupled dynamics, while real-time sensing helps morphing structures adapt to on-the-fly load changes. Stable morphing operations require a balance of elasticity, strength, and damping—a design principle also observed in natural flyers and swimmers [73].

#### 3.2.1. Coupling Between Flexible Structures and Fluid Environments

**Figure 7:**
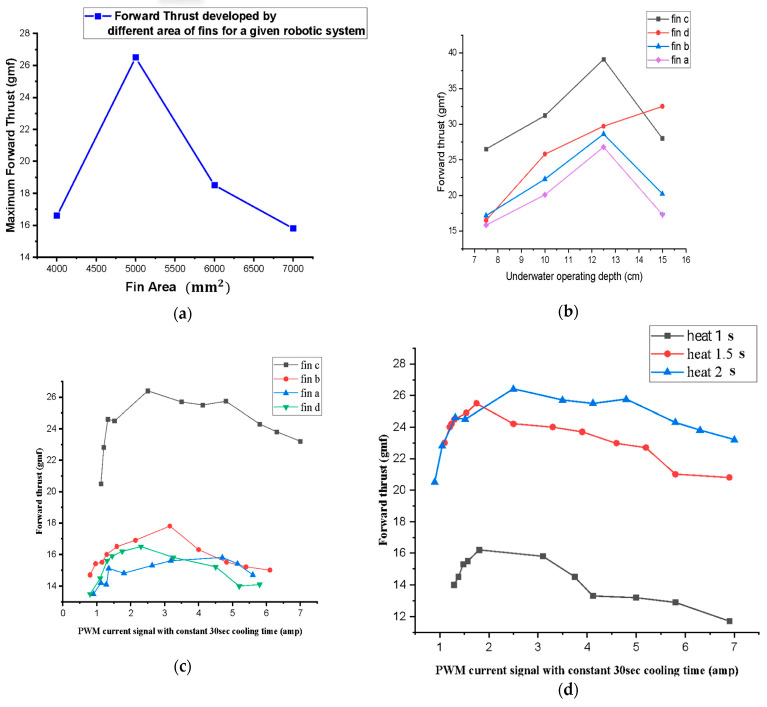
The relationship between thrust performance and key design variables in a flexible fishtail robot. (**a**) Optimal caudal fin area for maximizing forward thrust. (**b**) Impact of underwater operating depth on thrust for various fin geometries. (**c**) Influence of PWM-driven actuation current amplitude on forward thrust for different fin designs. (**d**) Effect of heating durations on propulsion efficiency, highlighting the importance of timing control in actuator performance(reproduced with permission from [75]).

In conventional aerospace or marine engineering, structures are typically considered rigid. Bioinspired morphing requires considering wings, fins, blades, or entire bodies as deformable elements. The local fluid pressure results in deflections, which alter boundary conditions in the flow [74]. This triggers a self-reinforcing loop: the modified shape alters the flow, which in turn alters structural deflection. Figure 7 illustrates that thrust first increases and then plateaus when the caudal fin grows beyond roughly 5000 mm^2^, reaches a depth-specific maximum for each fin geometry, rises with greater PWM current amplitude until performance saturation and eventual decline, and benefits from extended heating pulses only up to about 1.5 s, after which slower thermal cycling reduces propulsion efficiency. Fish fins exemplify this synergy:

*Shape memory alloy (SMA) spring actuators have been integrated into carangiform-inspired robotic fishtails to achieve efficient, bioinspired propulsion through flexible body deformation. By tuning parameters such as fin geometry, actuation current, PWM signals, and submersion depth, a hybrid caudal fin with a 5000 mm^2^ surface area generated a peak thrust of 40 gmf at 12.5 cm depth, demonstrating effective fluid–structure interaction [75].*

#### 3.2.2. Passive vs. Active Flow Adaptation Inspired by Nature

Some organisms, such as bats that adjust their wing tension or dolphins that modify skin compliance, actively change their form. Others passively interact with their environment, such as flexible fish fins that bend under currents [76].

In marine contexts, dolphins or whales might passively control flukes while actively adjusting flipper orientation. Both strategies inspire designs for robotic swimmers or morphing turbine blades that adapt passively under certain load ranges but also accept active control for more precise manipulation [77,78,79].

#### 3.2.3. Influence of FSI on Structural Integrity and Performance

Balancing elasticity, strength, and geometry carefully is crucial to avoid failures such as fluttering or buckling [80]. Unlike biological tissues that can self-repair, engineered morphing skins face durability challenges [81]. Similarly, animals often use distributed muscle actuation to avoid overstressing any one segment. Engineered systems can achieve a similar feature by localizing actuation across segmented or modular components [82].

### 3.3. Aerodynamic Phenomena in Morphing Systems

Bioinspired morphing structures in aerospace must address core aerodynamic phenomena such as vortex formation, stall management, unsteady flows, and the interplay of flexibility with lift/drag performance.

Nature-to-Device Bridge: *Flying organism wing leading-edge slats inspire adaptive flaps managing dynamic stall.*

#### 3.3.1. LEV

LEVs form when flow rolls up around a sharp or flexible leading edge in flapping or highly swept wings. Insects such as hawkmoths and fruit flies rely on stable LEVs to sustain high lift coefficients. Birds and bats also manipulate partial LEV formation using active or passive wing modifications [83].

**Figure 8:**
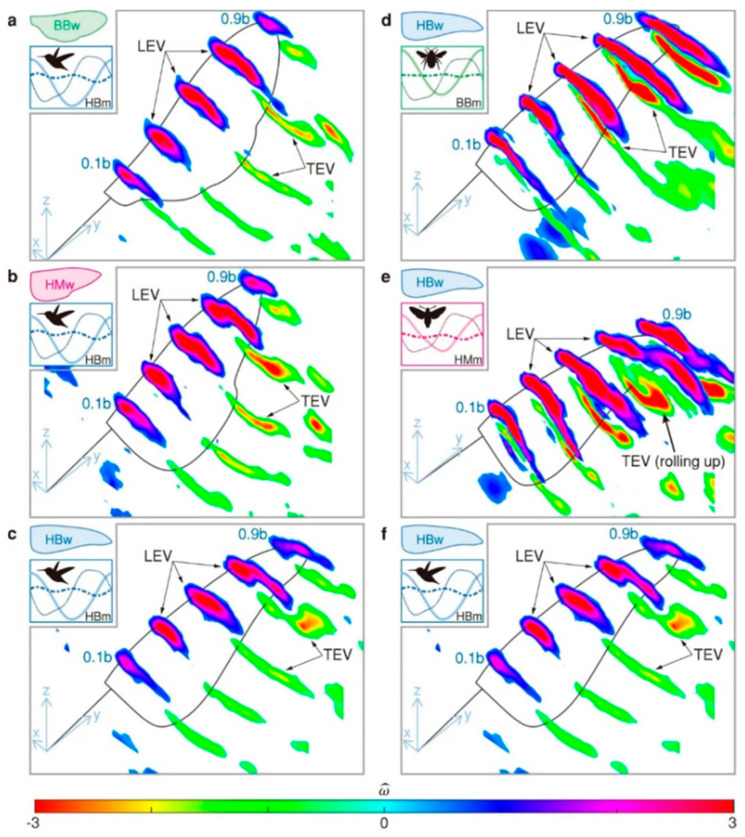
DPIV measurements showing vortex structures during the mid-downstroke phase, captured at various wing sections. Non-dimensional vortex strength ranges from −3 to 3, based on vorticity, average wing chord length, and wing tip speed. Comparisons include three wing shapes under the same flapping motion (hummingbird motion at (*t*/*T* = 0.26) and three flapping motions with the same wing shape (bumblebee at *t*/*T* = 0.30, hawkmoth at *t*/*T* = 0.24). Panels (**a**–**c**) correspond to the bumblebee, hawkmoth, and hummingbird wing shapes, respectively, all executing the hummingbird motion, whereas panels (**d**–**f**) illustrate the bumblebee, hawkmoth, and hummingbird motions, respectively, applied to the hummingbird wing shape. (reproduced with permission from [84]).

*This interplay between structural flexibility and unsteady aerodynamics has inspired the design of flapping-wing MAVs that attempt to replicate insect flight efficiency. Figure 8 (ref. [84]) illustrates the vortex structures observed during the mid-downstroke phase, highlighting variations across different wing shapes and flapping motions [84]. By incorporating flexible wing materials and carefully tuned mass distribution, engineers can reproduce LEV formation and harness the associated lift. However, controlling these unsteady flows in real time remains a significant challenge [85].*

While direct biomimicry of muscle-powered flight at aircraft scale has remained infeasible—analytical scaling laws show that specific muscle power falls two orders of magnitude short above ≈1 m span [86,87], the aerodynamic and structural principles of insect wings are highly effective for micro-aerial vehicles (MAVs, Re ≈ 10^2^–10^4^) and small-scale robotics. Low-Reynolds-number vortex control, compliant vein–membrane hinges, and span-wise flexion have yielded lift coefficients up to 1.6 in sub-gram flappers [88]. The same compliant-hinge concepts now enable exoskeleton finger joints that transmit 30% more torque per gram than pin-joint designs [89].

#### 3.3.2. Stall and Flow Separation Control

Birds reconfigure their wings, spreading or tilting their feathers to reduce local angles of attack. Albatrosses manipulate wing camber to maintain efficient soaring, and eagles use slotted wing tips for slower speeds without stalling [90].

*Feather Articulation and Slotting: Similar principles have been explored in engineering contexts. Winglet designs on aircraft wings mimic the function of reducing induced drag by controlling vortex formation. However, nature allows dynamic, real-time adjustments. Accordingly, feathers function like a distributed system of micro-control surfaces, each capable of slight but critical movements to fine-tune flight performance [91].*

Application to UAVs, MAVs, and Aerospace Designs

*In engineering, lessons from avian flight can translate to more fuel-efficient aircraft that adapt wing structures in response to varying flight conditions (take off, cruise, landing). Similarly, the concept of dynamic soaring has been studied for UAVs that can harness wind gradients for propulsion, reducing power consumption [92].*

Swifts’ ability to alter their wing sweep inspires variable-sweep wing aircraft. Dynamic camber control in birds parallels morphing airfoil designs where actuators or smart materials replicate discrete changes in airfoil shape [93].

#### 3.3.3. Dynamic Stall and Unsteady Aerodynamics

Dynamic stall, which frequently occurs in rotorcraft or flapping wings, emerges during rapid changes in angle of attack. Large transient loads can damage structures. Birds manage unsteady gusts partly by folding wings or adjusting covert feathers to minimize local flow separation. Active leading-edge devices or flexible chords can reduce dynamic stall, similar to how animals adapt to sudden speed changes. Testing with morphing rotor blades has demonstrated that modest geometry changes can alleviate dynamic stall vortices, improving aerodynamic efficiency [94].

### 3.4. Hydrodynamic Phenomena in Morphing Systems

In aquatic environments, morphing is crucial for drag reduction, efficient propulsion, and maneuverability in dense fluid media.

Nature-to-Device Bridge: *Aquatic appendage flexibility guides compliant propulsors, improving hydrodynamic thrust efficiency.*

#### 3.4.1. Vortex Dynamics and Bioinspired Propulsion

Fish exploit vortex shedding from bodies or fins to generate thrust with minimal energy cost. Flying fish even transition briefly into the air, morphing fins into quasi-wings.

*Fish fins containing flexible fin rays of tunable stiffness are of substantial interest to engineers seeking to develop bioinspired propulsors for underwater vehicles. An underwater drone could adapt fin stiffness in response to flow conditions, improving thrust and efficiency [95].*

#### 3.4.2. Flow Separation and Drag Reduction

Dolphins reduce friction drag through compliant skin structures, and humpback whales employ tubercles to mitigate stall.

*One of the more direct translations of cetacean-inspired morphing to engineering is the application of tubercles on wind turbine blades, aircraft wings, or hydrofoils. While tubercles themselves do not always represent a dynamic morphing mechanism, they can be part of a broader adaptive system if integrated into flexible leading edges [96].*

Engineering solutions are developed to effectively reduce drag and noise

*From an engineering perspective, a flexible, sensor-laden ‘smart skin’ that reduces drag or mitigates flow separation could substantially enhance the performance of AUVs or submersibles. Integrating this type of skin with dynamically adjustable internal support structures could emulate certain functionalities observed in cetaceans.*

These systems use flexible polymer matrices and braided giant magnetoresistance sensors (1D bending radius < 5 mm) that withstand 10,000+ bending cycles. The soft, shape-changing bodies of cephalopods similarly inspire soft robotics for stealthy, maneuverable submersibles. As presented in Table 1, Marine Skin v2 supports multi-parameter sensing up to a depth of 2000 m, making it ideal for deep-sea exploration.

### 3.5. Energy Efficiency and Elastic Energy Storage in Morphing

From flying birds to schooling fish, nature exemplifies how morphological alterations can harvest or conserve energy.

#### Principles of Elastic Energy Storage and Recovery

Biological structures often incorporate elastic tendons or spring-like tissues that store kinetic energy in one phase of motion and release it in another. Engineers replicate this using SMAs, flexible beams, or bistable composites:

*In fish, the interplay of body bending frequency, amplitude, and stiffness distribution ensures minimal wasted energy in wave propagation. Evolution has converged on optimum designs for flexible fins that resonate with typical swimming speeds. This phenomenon resembles mechanical resonance, where minimal input is required to sustain large oscillations.*

Insect flight muscles operating near resonance, fish harnessing Kármán vortices, or birds exploiting dynamic soaring all demonstrate ways of conserving net energy. Engineers attempt analogous approaches in flapping-foil energy harvesters, wave-energy converters, or morphing rotor concepts. Practical demonstrations range from partial morphing aircraft flaps that conserve strain energy to robotic fish employing soft polymer spines:

*Morphing UAV prototypes demonstrate up to 30% improvements in flight endurance compared to similar-weight, rigid-wing drones. Meanwhile, flexible-fin underwater robots have recorded up to 20% improvements in thrust efficiency.*

### 3.6. Stability, Control, and Adaptive Response

Beyond shape adaptation, the integrated sensory and control loops exhibited by organisms are a key feature of bioinspired morphing. Birds rely on feather proprioceptors, fish on lateral lines, and bats on sensory hairs to facilitate quick reflexes. Engineers mimic this distributed approach with local sensor-actuator loops:

*Recent flapping-wing UAVs embed arrays of strain or pressure sensors within their compliant membranes; these local signals drive neighboring tendon actuators in millisecond-scale loops, mirroring the distributed proprioceptive feedback of bat wings and allowing rapid disturbance rejection without burdening a central processor [100].*

While morphing can enhance performance, it also introduces complexities like flutter or divergence. Although biological tissues self-repair and adapt, engineered morphing skins need to withstand repeated deflections without fatigue or delamination. Hence, technological challenges remain, including scaling, durability, regulatory certification, and cost. Yet successful prototypes, e.g., shape memory alloy, driven aircraft flaps, or robotic fish with embedded muscle-like actuators, demonstrate the feasibility and promise of bioinspired morphing.

Summarized in Table 2, the biological inspiration morphing concepts mapped to their engineering analogues, together with the key performance metrics, innovation potential, and overall outcome (Successful/Partial).

## 4. Bioinspired Morphing in Aerodynamics

Bioinspired morphing has emerged as a foremost dynamic frontier in aerodynamic research, promising to enhance flight efficiency, maneuverability, and versatility across a wide range of applications, from large commercial airliners to UAVs [101,102]. Conventional aircraft designs have long relied on fixed aerodynamic surfaces optimized for a limited set of conditions (e.g., cruise flight), which often results in performance compromises at off-design states such as takeoff, landing [103]. In contrast, natural flyers—birds, bats, and insects—routinely adapt their wing shapes in real time, tuning lift, drag, and control authority to suit rapidly changing flight requirements. This capacity for continuous or discrete reshaping, often referred to as morphing, allows organisms to achieve flight efficiencies and maneuverability significantly superior to a purely rigid wing system [104].

Inspired by these natural solutions, engineers have developed a wide array of morphing technologies to replicate or approximate biological adaptability [105]. Aeronautical designers can create systems capable of on-demand adjustment of their aerodynamic characteristics by incorporating principles derived from flexible feather arrangements, membrane wings, flapping motions, and variable-camber profiles observed in biological flyers. Such systems promise multiple advantages: fuel savings via better lift-to-drag ratios at different flight regimes, noise reduction by mitigating turbulent flow structures, improved handling qualities in gusty or turbulent conditions, and expanded mission envelopes for both manned and unmanned flight [106]. Subsequent sections delve into the key areas where bioinspired morphing is reshaping aerodynamics, covering morphing wing technologies for large aircraft, the role of morphing in drag reduction and lift enhancement, applications in flapping-wing UAVs and rotorcraft, and the materials and control systems underpinning these innovations. We will examine how nature’s adaptive strategies continue to guide the design of shape-changing structures and how ongoing research addresses the practical challenges of integrating morphing into actual aeronautical systems.

### 4.1. Bioinspired Mechanisms for Optimizing Aerodynamic Forces

Nature-to-Device Bridge: *Avian wing morphing informs adaptive aerofoils optimizing lift-to-drag across regimes.*

#### 4.1.1. Variable Camber and Real-Time Optimization

A core aerodynamic benefit of morphing is the ability to reduce drag and increase lift over a broad range of operating conditions [107]. Conventional airframes often compromise between low-speed and high-speed performance, leaving the aircraft suboptimal for large portions of the flight envelope. However, birds seamlessly transition between gliding, climbing, diving, and tight maneuvering by adjusting their wing shape, a universal adaptability that engineers attempt to emulate [108].

- Low-Speed Regimes: During takeoff and landing, an aircraft with morphing capabilities could increase wing camber to generate higher lift, reducing runway length and approach speed. This mirrors birds that fan out their primaries and increase their wing area for better lift at low speeds. Once at a given altitude, the wing could flatten or reduce its camber for a more aerodynamic profile, lowering drag and conserving fuel [109]. Large soaring birds like albatrosses do something similar by locking their wings in a slender, high-aspect-ratio configuration to glide over oceans with minimal energy expenditure.
- Real-Time Optimization: Rapid changes in wing twist or leading-edge shape can help fighters or agile UAVs maintain optimum lift at high angles of attack. Raptor-like dynamic twisting of wingtips allows tight turns or stoops. Real-time optimization loops, often employing CFD coupled with control algorithms, can continuously seek an optimal shape based on sensor feedback (airspeed, angle of attack, load factor), analogous to how birds sense local airflow and respond via muscle forces [110].

#### 4.1.2. Flow Control and Vortex Management

Beyond adjusting lift or drag coefficients, morphing can also serve advanced flow-control functions. Animals like bats control their local membrane tension to manage LEVs, while insects generate and exploit transient vortices for higher lift [111,112]. In a high-angle-of-attack flight, a strong LEV can delay stall and boost lift, and a morphing leading edge that actively controls curvature or surface roughness can influence vortex formation. Continuous morphing trailing edges can tailor the wake to reduce drag or re-energize boundary layers [112]. Some designs feature slits or channels that adjust in response to flight conditions, allowing the manipulation of airflow. By embedding sensor arrays (pressure sensors, strain gauges, or MEMS-based flow sensors) in the morphing surfaces, the system can detect incipient flow separation or unfavorable vortices and adjust within fractions of a second, maintaining aerodynamic stability [113].

#### 4.1.3. Noise Reduction

A lesser-discussed but important aspect of aerodynamic morphing is noise mitigation. Birds like owls have specialized wing features—serrated leading edges and downy trailing edges—that reduce turbulence-induced noise, allowing nearly silent flight [114,115]. Translating these principles into morphing aircraft could benefit commercial aviation, especially in mitigating airport noise pollution.

**Figure 9:**
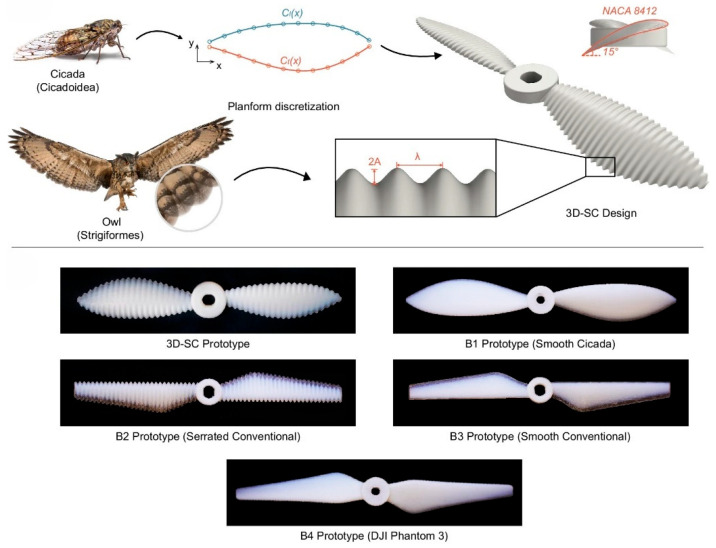
Sequential development of the 3d-sc propeller topology inspired by owl feathers and cicada forewings, ending with a cad representation. The inset defines the sinusoidal serration waveform’s wavelength and amplitude, along with the 2D airfoil profile (reproduced with permission from [115]).

Figure 9 (ref. [115]) illustrates the design and fabrication process of the 3D-SC propeller. The top panel presents the sequential development of the propeller topology, inspired by the morphology of owl feathers and cicada forewings, closing in a CAD model. The inset provides details on the sinusoidal serration waveform parameters (wavelength and amplitude) and the airfoil profile. The bottom panel presents the fabricated 6-inch diameter propeller alongside benchmarks (B1–B4), produced using Poly-Jet additive manufacturing to demonstrate the progression from conceptual design to physical prototype. Deployable or morphing serrations on leading or trailing edges can break up large turbulent eddies into smaller, less intense structures, minimizing tonal noise. Thinning or thickening the trailing edge at different flight phases can decrease overall broadband noise. Because noise generation depends on local flow conditions, real-time morphing surfaces can readjust and minimize vortical structures or flow separation around wing flaps, slats, or landing gear elements [114]. Despite historically being a subordinate design consideration to performance and safety, acoustic mitigation is gaining prominence as a significant research avenue in morphing technologies, driven by increasingly stringent regulations and environmental imperatives.

### 4.2. Flapping Wing UAVs and MAVs: Bioinspired Approaches

Although large-scale morphing aircraft attempt to optimize lift-to-drag in mostly fixed-wing configurations, flapping wing UAVs or MAVs draw even more directly from biology.

Nature-to-Device Bridge: *Flapping wings inspire MAVs to achieve lift-thrust coupling* via *unsteady aerodynamics.*

Beyond wing morphing, bats and insects [116] employ flapping flight, a sophisticated mechanism that simultaneously generates lift and thrust through complex unsteady aerodynamic principles. Aerodynamic modeling of bat-inspired flapping robots utilizes SMAs for muscle-like actuation [117]. The engineering replication of these principles has inspired a distinct subfield of bioinspired aerodynamics, centered around small drones that can hover, maneuver in tight spaces, and perch on surfaces with high efficiency.

#### 4.2.1. Insect-Inspired Flapping

Several insects (e.g., bees, dragonflies, butterflies) rely on rapid wingbeats that generate and manage LEVs [118]. By flapping, rotating, and twisting their wings at precise angles, insects exploit unsteady flow phenomena to produce high transient lift forces.

Pioneering research at institutions like Harvard has produced small, lightweight robots with piezoelectric actuators driving flapping wing motions. These micro-scale robots can achieve basic flight; nevertheless, challenges remain in power storage, stable control, and real-time LEV manipulation [119].

**Figure 10:**
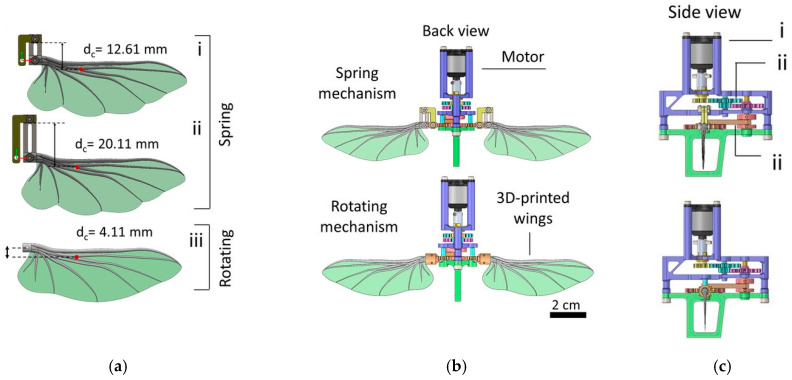
(**a**) Axes of rotation for leaf spring and rotating wings. (**b**) Back views of flapper mechanisms with spring (top) and wing rotation (bottom). (**c**) Side views of the mechanisms (reproduced with permission from [120]).

Insects often have thin, flexible membranes with reinforcing veins. These membranes can passively deform under aerodynamic loads, improving efficiency. Synthetic analogs use thin polymer films with integrated carbon-fiber frames or micro-actuators that replicate vein function. Some MAVs mimic the wing’s passive flexibility and active rotation, modulating wing orientation to adapt to forward flight, hovering, or turning. This can significantly improve stability in gusty or cluttered environments, similar to a housefly’s ability to navigate dense obstacles [120]. Figure 10 presents the mechanical design of the flapping mechanism. Figure 10a highlights the axes of rotation for two pitching mechanisms: leaf spring wings and rotating wings, featuring their shortest (6 mm) and longest (13.5 mm) springs. Figure 10b illustrates the color-coded back views of the flapper with spring and wing rotation mechanisms, while Figure 10c presents the corresponding side views for better visualization.

#### 4.2.2. Bat-Inspired Membrane Wings

Bats exhibit a distinct model: their wing membranes span elongated finger bones, enabling large, continuous deformations. Bat wings can substantially alter their camber, twist, and planform area during flight. A framework of lightweight rods (representing bat digits) is covered by a stretchable membrane [121]. By pulling on different rods with cables or shape memory actuators, the membrane’s camber is altered. Some prototypes incorporate active materials within the membrane to vary stiffness locally, similar to how bats retract or relax parts of the membrane. This allows discrete control over aerodynamic forces, especially helpful during maneuvering or gust response [122]. Bat-inspired UAVs can theoretically outmaneuver rigid drones, especially in indoor or urban canyons. Their flexible wings can absorb and adapt to sudden wind gusts, similar to how bats fly effortlessly in complex environments.

Beyond pure aerodynamics, the large membrane surfaces can also serve other functions, such as mounting solar cells for energy harvesting or integrating sensory arrays that detect collisions or measure airflow. This mirrors biological multifunctionality, where bat wings play roles in thermoregulation, tactile sensing, and even social communication.

#### 4.2.3. Bioinspiration from Birds, Bats, and Insects

Though birds do not rotate their wings continuously like a helicopter, some do incorporate cyclical flapping patterns reminiscent of rotating blade dynamics in a single revolution. Insects with leading-edge vortex generation during flapping might inspire advanced rotor designs that exploit unsteady aerodynamic lift. Conceptually, a rotor blade could intentionally promote and manage LEVs on the advancing side to boost lift. Although such approaches remain experimental, they demonstrate the potential for improved hover efficiency or forward speed capabilities.

Compared to fixed-wing flight, flapping can be energy-intensive, especially if not optimized for resonance or if the mass of actuators is significantly high. Several insects rely on specialized flight muscles and hinge thoracic structures that store and release elastic energy. Replicating this at scale requires advanced materials and intelligent structural design. Generating stable flapping motion requires precise synchronization of wing kinematics in multiple degrees of freedom. Small deviations can result in rapid loss of control, especially at micro scales. As aforementioned, insect wings can self-heal or regrow to some extent, but synthetic materials wear down over repeated high-frequency flapping cycles. A pressing issue is the identification of robust, lightweight materials capable of withstanding tens of thousands of flap cycles. Furthermore, simple scaling of wing flapping does not always hold as MAVs increase in size. The effects of the Reynolds number can alter the flow regime, requiring design modifications. Conversely, extremely small flapping vehicles confront aerodynamic phenomena like low Reynolds number flows with high viscous effects. Nevertheless, the potential payoff, hovering with bird-like, bat-like, or insect-like agility, perching capabilities, and improved gust tolerance, ensures continued research into flapping wing morphing vehicles. Several breakthroughs in micro-actuation, flexible materials, and real-time control are likely to cross-pollinate with larger-scale morphing aircraft designs.

**Figure 11:**
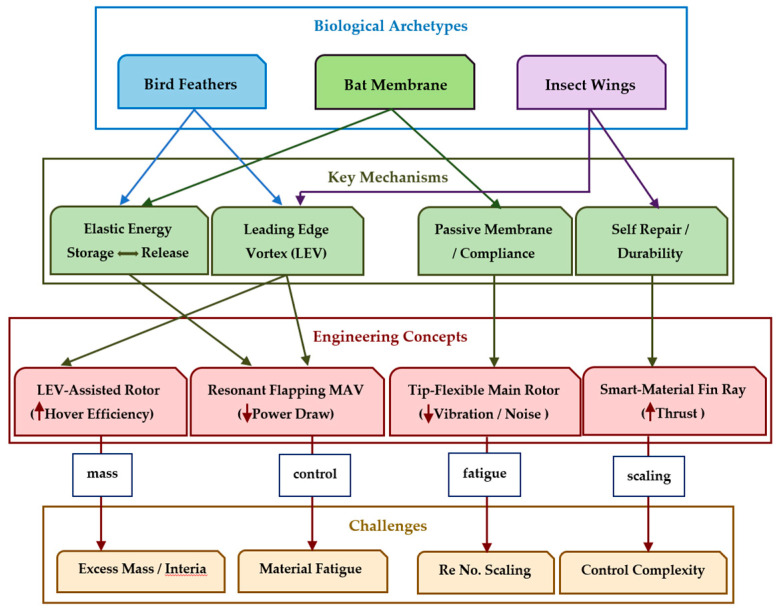
Bio-inspiration pathway from flapping animals to tunable-stiffness propulsors. Three biological models supply four core mechanisms—leading-edge-vortex lift, elastic energy recycling, passive membrane compliance, and self-repair—that inform emerging rotor, wing, and fin concepts. Promised gains include higher thrust, lower power demand, reduced vibration, and quieter operation, but challenges remain in mass, fatigue, Reynolds-number scaling, and control authority. Arrows trace the stepwise influence from biological archetypes (top) through mechanisms, concepts, and finally their challenges (bottom); within the concept boxes, an upward arrow (↑) signifies a desired increase, while a downward arrow (↓) signifies a desired decrease.

Furthermore, flexible membrane wings observed in bats or large insects might guide future rotor designs where certain portions of the blade can passively deflect under load, tuning the local angle of attack. While full-scale membrane rotors might be impractical, partially flexible sections near the tips could dampen vibrations and reduce rotor noise, similar to how whale flippers with tubercles can reduce stall onset. The translation of biological flapping mechanisms into tunable-stiffness engineering designs is illustrated in Figure 11.

### 4.3. Morphing Wing Technologies for Aerospace Applications

For decades, aircraft designers have experimented with variable geometry to broaden operational envelopes. Early examples include variable-sweep wing fighters, such as the F-14 Tomcat or Panavia Tornado, which shift wing sweep angles in flight to balance low-speed lift and high-speed drag reduction. Although effective, these mechanical solutions rely on large hinges, heavy actuators, and discrete segments that create aerodynamic discontinuities (gaps and steps). However, from a bioinspiration perspective, the ideal is a smooth, continuous surface that seamlessly changes its shape without compromising aerodynamic integrity [2].

Natural flyers exemplify such seamless adaptivity: birds bend their joints and adjust individual feathers to fine-tune wing shape, while bats deform their membrane wings to generate precise lift and thrust profiles.

Nature-to-Device Bridge: *Feather articulation inspires continuous morphing wings, ensuring smooth shape adaptation.*

Inspired by these observations, engineers have increasingly focused on continuous morphing concepts. Instead of hinged flaps, morphing wing designs employ compliant materials, distributed actuation systems, and flexible skins to gradually reconfigure airfoil shape (camber, thickness, and twist) or planform geometry (span and sweep) [2]. This enables more refined aerodynamic control with fewer drag tradeoffs and mechanical complexities compared to conventional hinged mechanisms.

#### 4.3.1. Adaptive Camber and Variable Thickness

One of the simplest yet most impactful forms of morphing is the ability to adapt an airfoil’s camber or thickness distribution in real time. By adjusting the camber, an aircraft can increase lift during takeoff or maneuvering, then reduce drag in cruise. Birds achieve similar adjustments by controlling feather angles and tension in wing musculature. Accordingly, engineers have explored various smart materials and structural designs [123].

**Figure 12:**
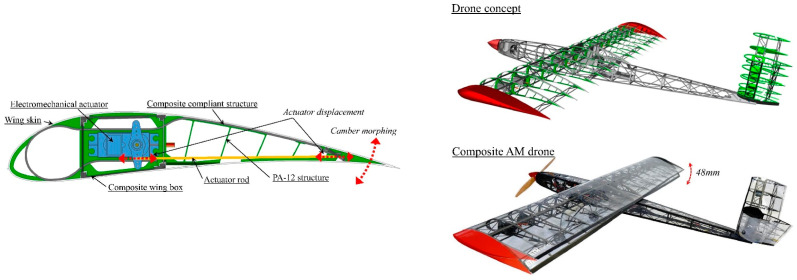
Camber morphing drone using compliant composite structure and electromechanical actuators. Right Top: Concept model with truss fuselage, morphing ribs, and wing tips. Right Bottom: 3D-printed prototype demonstrating actuation (reproduced with permission from [126]).

Some morphing wings incorporate honeycomb-like internal structures bonded to flexible skins. Actuators (e.g., pneumatic, hydraulic, or shape memory alloy-based) expand or contract the honeycomb cells, resulting in a smooth outer surface that alters curvature without forming discrete hinges. Instead of rigid spars, the internal load-bearing structure can comprise compliant mechanisms, specialized linkages, or elastically deformable beams that bend predictably under actuator input. Aircraft skins must balance flexibility (for shape change) and rigidity (to handle aerodynamic loads). Engineers have tested elastomeric skins, corrugated composite skins, or fiber-reinforced elastomeric skins, each designed to stretch, shear, or bend in controlled ways while resisting in-plane loads [124,125]. This concept is illustrated in Figure 12 (ref. [126]), which illustrates a camber morphing drone integrating composite structures and electromechanical actuation [126].

Such morphing airfoils can also exhibit variable thickness, adjusting the maximum chordwise depth or its fore-aft location. Airfoils with increased thickness can generate greater lift at lower velocities, whereas thinner profiles diminish drag at higher velocities. Although these shape alterations are often subtler than dramatic wing sweeps, they can yield significant performance improvements over a flight mission.

#### 4.3.2. Adaptive Winglets and Leading-Edge Devices

**Figure 13:**
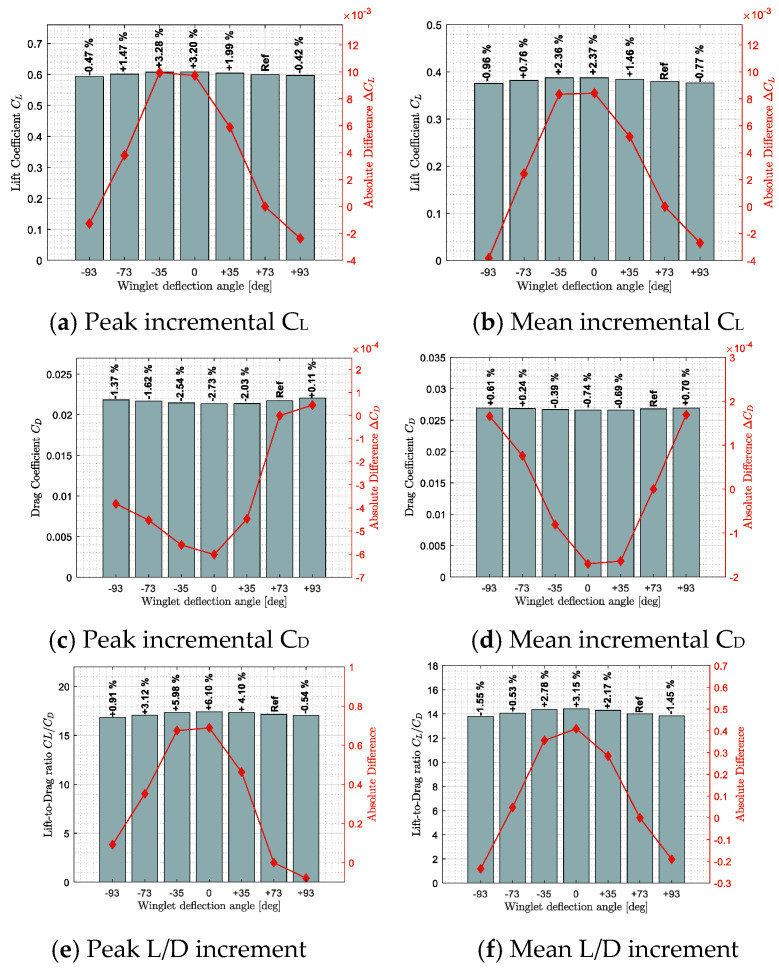
Aerodynamic pay-off from an actively deflecting winglet installed on a CRJ700 across the range ξ = −93° to +93°; (**a**,**b**) Incremental lift coefficient: grey bars give the peak value among 16 climb/cruise cases, red curve the corresponding mean. (**c**,**d**) Incremental drag coefficient shown in the same format; (**e**,**f**) Resulting lift-to-drag ratio enhancement. The best compromise appears near ξ ≈ −35°, where lift rises by ~3%, drag falls by ~2.7%, and the lift-to-drag ratio improves by ~6%. (Reproduced with permission from [127]).

Wingtip devices, such as winglets or sharklets, reduce induced drag by mitigating the strength of wingtip vortices, yet most conventional winglets are fixed. Fixed winglets suppress induced drag, but they cannot reshape themselves the way birds continually fan or tuck their primary feathers. Recent prototypes therefore pursue adaptive winglets whose tips fold, twist, or change dihedral—some even embed piezo-electric layers for real-time twist control. Flight tests on a Bombardier CRJ700 equipped with such a system report that a −35 ° deflection yields roughly 3% more lift, 2.7% less drag, and a 6% lift-to-drag gain (Figure 13). The data is reproduced from [127], providing the performance benchmark for the design envelope assessed.

Leading-edge devices like slats conventionally improve low-speed lift but add weight, complexity, and drag when deployed. Bioinspired approaches attempt to create a continuously deformable leading edge, similar to how bird feathers [128] or bat wing membranes [129] shape themselves to maintain desirable flow attachment. NASA and FlexSys Inc. (Arbor, MI, USA) [130] conducted extensive flight tests of a morphing trailing edge system, but conceptually similar approaches also apply to leading edges. By carefully shaping the leading edge, engineers can delay flow separation and enhance lift or reduce drag, depending on the flight regime. Owls adopt serrated leading edges to minimize noise. Translating serration patterns to aerodynamic leading edges can also provide boundary-layer control [114]. Combined with a morphing capability, these serrations might enlarge or shrink to optimize flow laminarity or minimize acoustic footprints.

### 4.4. Materials, Actuation, and Control in Aeromechanics Morphing Systems

Morphing aircraft rely significantly on materials that can flex, bend, and recover repeatedly without compromising structural integrity.

Nature-to-Device Bridge: *Muscle-like actuation inspires shape-memory alloys powering precise morphing control systems.*

In nature, feathers or membranes combine lightness, elasticity, and self-repair mechanisms honed over millennia. Engineers are yet to replicate these properties comprehensively; nevertheless, continuous progress in smart materials and composites narrows this gap.

#### 4.4.1. Smart Materials

Nitinol and related alloys can revert to a “memorized” shape when heated above their transformation temperature. They generate high forces for their weight, making them desirable for morphing applications [131]. However, SMAs can be slow to cool, limiting actuation frequency. Materials like PZT (lead zirconate titanate) or PVDF (polyvinylidene fluoride) deform under voltage. Thin piezoelectric patches integrated into wings can produce negligible shape alterations at high frequencies, useful for flutter suppression or micro-adjustments [132,133]. Sometimes called “artificial muscles”, EAPs can exhibit significant strain when subjected to an electric field. They are categorized into dielectric elastomers, ionic polymer-metal composites (IPMCs), and conductive polymers. Challenges include high voltage requirements (for dielectric elastomers) or low force output (for IPMCs) [134]. Alloys like Terfenol-D expand or contract in response to magnetic fields. While they can generate large forces, they often require substantial magnetizing coils and are comparatively heavy. By pumping fluid or air into embedded channels, engineers can create pneumatic artificial muscles or soft actuators. These can generate significant shape alterations with potentially smooth, continuous motion, though the fluid supply increases mass and complexity.

Nature’s materials often serve multiple roles—structure, actuation, sensing, and even self-healing. Engineering researchers attempt to emulate such multifunctionality by embedding conductive filaments or piezoresistive networks in a composite wing skin to provide real-time strain or deformation data, mimicking the role of mechanoreceptors in bird feathers. Negligible cracks or punctures can compromise morphing surfaces. Advanced polymers that chemically bond or physically reflow at damaged sites could restore structural integrity, reducing maintenance costs. Some shape memory or piezoelectric elements can harvest vibration energy to power sensors or offset actuation demands, emulating the way biological muscles and tissues can reclaim energy during cyclical motion (e.g., tendon elasticity in bird wings).

#### 4.4.2. Distributed Actuation Strategies

A key enabling technology in morphing wing systems is distributed actuation, wherein several small actuators, embedded in the wing’s structure or skin, work cohesively to bend or twist local sections. This parallels the distributed muscular control observed in birds and bats, allowing localized shape alterations rather than a single uniform transformation. Potential actuator technologies include:

- SMAs: Alloys like NiTi (Nitinol) can alter their shape when heated above a certain threshold. By embedding SMA wires or ribbons into a flexible wing structure, engineers can achieve localized deformations. Although SMAs are lightweight, they can be slow to cool down and require careful thermal management [124,135].
- Piezoelectric Actuators: Piezoelectric patches convert electrical signals into mechanical strain rapidly. While they produce negligible displacements, attaching them in an amplified or leveraged configuration can yield larger shape alterations. They are often desirable for high-frequency or small-amplitude shape corrections, useful in active flutter control [136,137].
- EAPs: These are polymers that deform significantly under an electric field. They can mimic muscle-like contractions, making them desirable for biomimetic wing designs, especially in smaller drones or MAVs [133].
- Hydraulic or Pneumatic Networks: Inspired by animal blood flow or cephalopod hydrostatics, flexible channels in a wing can inflate or deflate, causing the wing to bulge or flatten. While potentially heavier than other options, such fluidic systems can achieve robust, continuous deformations over large areas [138].

These distributed actuators require robust sensing, feedback control, and power distribution. The synergistic integration of actuators, sensors, and flexible structures, when appropriately implemented, yields a morphing wing that continuously adapts its shape to optimize aerodynamic efficiency under dynamically changing flight conditions.

#### 4.4.3. Challenges in Structural Integration

While materials technology advances steadily, integrating these smart materials into an airplane’s load-bearing structure is challenging. The addition of actuators, sensor networks, or fluid lines can significantly increase weight if not carefully managed [139]. A wing must remain strong enough to handle gust loads or emergency maneuvers. SMA-based systems require repeated heating and cooling; piezoelectrics undergo cyclical strain at possibly high frequencies [140,141]. Each cycle can induce micro-cracks or degrade structural integrity if not properly analyzed. Morphing surfaces must maintain aerodynamic smoothness, even under repeated deformation. Abrasion, weather effects, and UV exposure can degrade flexible skins over time.

Solutions that work well on small UAVs may struggle to scale up to commercial airliner wings owing to drastically higher loads and stricter regulatory standards. Given these challenges, several current morphing concepts target smaller or specialized aircraft, such as UAVs, where certification hurdles and payload constraints are more manageable. Nonetheless, incremental adoption of morphing components (e.g., adaptive wingtips or trailing edges) in larger aircraft is currently being studied [140].

#### 4.4.4. Control and Sensing in Aerodynamic Morphing

In addition to being a material or structural challenge, morphing is also a control problem. Dynamic shape alterations can radically alter aerodynamic coefficients, potentially leading to instabilities if not managed properly [142]. Natural flyers rely on distributed sensors—feather mechanoreceptors, wing membrane nerve endings, or insect cuticular hairs—to gauge local airflow, and then reflexively adjust wing shape via muscle contractions. Engineers are required to replicate these capabilities with sensor networks and real-time control algorithms [143].

Arrays of miniaturized pressure taps, or MEMS-based sensors, can measure local pressure distributions across the wing [144,145]. By analyzing differences in pressure, the control system infers lift, angle of attack, and incipient flow separation. Bonded to or embedded in the wing structure, these sensors detect bending, twisting, and tension [143,144]. They enable the control system to interpret the effectiveness of morphing adjustments. An inertial measurement unit (IMU) provides data on the aircraft’s angular rates, accelerations, and orientation, which is essential for flight stability and maneuvers [146].

Some advanced prototypes utilize optical flow measurements or miniature ultrasonic anemometers to detect boundary-layer transitions or vortex formations, enabling early corrective morphing actions.

#### 4.4.5. Real-Time Control Algorithms

Bio-inspired morphing requires closed-loop control. The system receives sensor inputs about aerodynamic conditions and structural states, processes these signals (often with high-speed microprocessors or field-programmable gate arrays), and outputs commands to distributed actuators. For simpler morphing tasks (e.g., trailing edge deflection), linear or quasi-linear control frameworks can suffice, especially if the shape alterations are modest [147]. Morphing significantly alters plant dynamics. Adaptive control algorithms can learn or update control parameters spontaneously to ensure stability despite varying wing geometry or external disturbances [148]. Emerging research employs reinforcement learning (RL) or neural networks to manage the high-dimensional space of morphing commands. An AI system can determine optimal wing shapes for various flight conditions by simulating or testing different morphing strategies. Model predictive control (MPC) utilizes predictive models of the aircraft’s response to evaluate future states under actuator commands, selecting the best control action. This is beneficial when abrupt, significant shape alterations risk overshooting or causing flutter [149]. A major challenge lies in the coupled fluid-structure dynamics: when a wing morphs, the aerodynamic forces shift, which further deforms the wing, altering the forces again. Accurate CFD modeling of these interactions in real time can be computationally heavy, necessitating advanced model reduction, fast surrogate models, or specialized hardware accelerators.

To explain the technological landscape, Table 3 classifies morphing systems by their primary aerodynamic role. This thematic structure enables direct comparison of the biological models, actuation strategies, and real-world implementations across multiple performance domains.

## 5. Bio-Inspired Morphing in Hydrodynamics

The vast expanse of Earth’s oceans, rivers, and lakes teems with organisms of diverse shapes and sizes exhibiting remarkable locomotion, maneuverability, and energy efficiency. A critical attribute underlying many of these capabilities, shared with nature’s aerial counterparts, is morphological plasticity: the ability to adjust bodily or appendage geometry in response to dynamic flow conditions. Mirroring the effortless wing reshaping of birds, fish, marine mammals, and cephalopods similarly alter their fins, flippers, flukes, and body contours to optimize propulsion or maneuvering in water.

For engineers, the marine environment presents both opportunities and challenges. Water is denser than air, allowing smaller devices (relative to mass) to generate significant thrust or lift. However, this density also translates into higher drag forces and a more significant risk of flow-induced vibrations or fatigue. Bioinspired morphing in hydrodynamics responds to these realities by emulating the adaptive mechanisms observed in aquatic species. The result is a growing body of work on flexible propulsors, morphing hydrofoils, shape-changing energy harvesting devices, etc. Together, these innovations attempt to expand the performance envelope of underwater vehicles, minimize energy costs in marine operations, and enhance the viability of renewable energy extraction from waves and currents.

Here, we examine how nature’s swimmers inspire hydrodynamic morphing, highlight core engineering approaches and materials, discuss the role of control systems and computational modeling, and explore the future of bioinspired aquatic design. We investigate morphing propulsors for marine vehicles, bioinspired hydrodynamic energy harvesting, CFD and experimental investigations, and prevailing engineering challenges. Hydrodynamic performance can be enhanced in submarines and tidal turbines by employing dynamic shape adjustment inspired by avian wing morphing.

**Figure 14:**
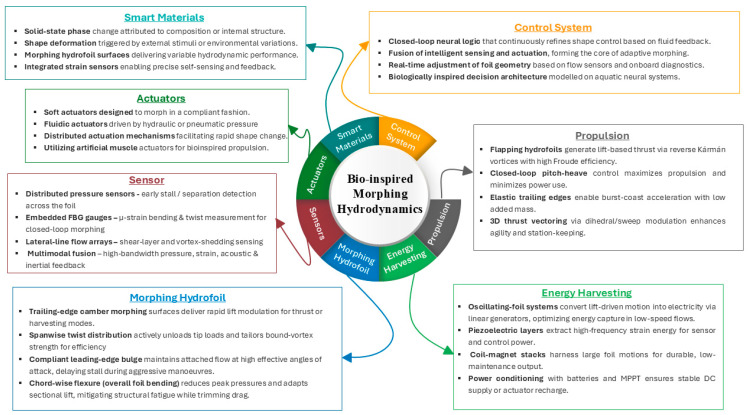
Radial infographic summarizing the interdisciplinary pillars of bioinspired morphing hydrodynamics. The central hub highlights the overarching theme, while six color-coded spokes depict (clockwise) smart materials, control system, propulsion, energy harvesting, morphing hydrofoil, sensors, and actuators. Each spoke pairs an icon set with key design notes shown alongside: adaptive materials enable shape change; closed-loop control links sensing and actuation; lift-based propulsion and energy-harvesting foils exploit unsteady vortex dynamics; hydrofoil morphing strategies tailor camber, twist, and flexure; multimodal sensors provide high-bandwidth feedback; and compliant or fluidic actuators realize the required deformations.

Figure 14 presents an integrated framework that positions bioinspired morphing hydrodynamics at the intersection of six mutually supportive research pillars. Smart materials provide the intrinsic capacity for shape alteration, enabling hydrofoils to adapt their geometry without incurring significant mass penalties. A closed-loop control system fuses real-time sensor inputs with actuator commands, continuously refining foil shape in response to flow conditions. When operated in propulsion mode, phase-optimized pitch–heave motions generate a reverse Kármán vortex street that generates high-efficiency thrust. The same unsteady lift forces can be redirected toward power capture in an energy-harvesting cycle using electromagnetic, piezoelectric, or hybrid transducers. The morphing hydrofoil itself is the geometric fulcrum of the framework. The dynamic modulation of camber, twist, and chordwise curvature stabilizes leading-edge vortices, controls stall, and supports structural loads. These shape alterations are realized by distributed, compliant actuators that mirror the flexibility of biological fins, while an array of multimodal sensors—pressure taps, strain gauges, and lateral-line-inspired flow probes—provides the high-bandwidth feedback required to synchronize material response, actuation, and control. Together, these six domains form an integrated design space for developing agile, efficient, and adaptive aquatic vehicles and energy devices.

### 5.1. Morphing Propulsors in Marine Vehicles

#### 5.1.1. Inspirations from Fish Locomotion

The most iconic symbol of hydrodynamic morphing is the fish fin. Rather than being rigid plates or unstructured membranes, fins exhibit a structure comprising flexible fin rays interconnected by thin membranes, allowing variable stiffness and curvature.

**Figure 15:**
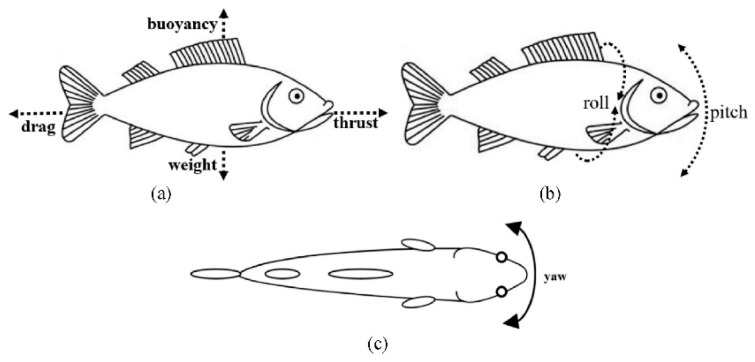
Definitions related to a fish’s (**a**) free body diagram and directional changes involving (**b**) Pitch, Roll, and (**c**) Yaw (Reproduced with permission from [150]).

Fish modulate fin shape and orientation to achieve multiple locomotor modes (e.g., cruising, sprinting, braking, and turning) with minimal energy expenditure. Figure 15 (ref. [150]) presents the free body diagram of a fish and its directional changes, including pitch, roll, and yaw.

##### Fin Ray Flexibility and Control

Each fin ray can bend due to a combination of passive elasticity and active muscular input. This bending reorients the fin’s surface relative to the incident flow, modulating wake vortex patterns to optimize thrust generation or maneuvering torque [151,152]. Embedding SMAs or EAPs in synthetic fin rays enables controlled deflection under electrical input. Artificial fin rays can be curved via cables or tendons running along their length, with springs or elastic elements facilitating return to a baseline configuration. Adjustable rods or fluid-filled channels enable switching the fin between stiff (high-speed cruising) and compliant (maneuvering) states [44].

##### Passive vs. Active Morphing

In many fish, fins deform passively under hydrodynamic forces to enhance beneficial bending, conserving muscular energy. Engineers also explore passive morphing––where a deformable section of a foil or hull flexes in response to fluid forces––to reduce flow separation or decrease drag without continuous actuation costs. In contrast, active morphing utilizes motorized or chemical stimuli to achieve deliberate shape alterations. Combining passive and active strategies can yield efficient, adaptive systems.

##### Robotic Fish and Underwater Drones

Laboratories worldwide have built biomimetic robotic fish that test morphing fin concepts:

*An early and successful bioinspired robotic fish employed segmented body sections actuated by servo motors. Although more similar to body undulation than fin morphing, it highlighted how continuous shape changes can replicate fish propulsion patterns [153]. Several prototypes include pectoral or caudal fins with embedded actuators that adjust fin shape in real time. The results are quieter operation (minimized propeller noise) and improved maneuverability. Certain fish rely on multiple fins (dorsal, anal, pectoral, pelvic). Similarly, engineers have explored vehicles with multiple morphing fins that coordinate for propulsion and posture control [154].*

**Figure 16:**
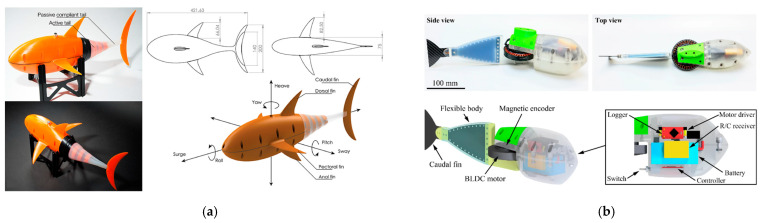

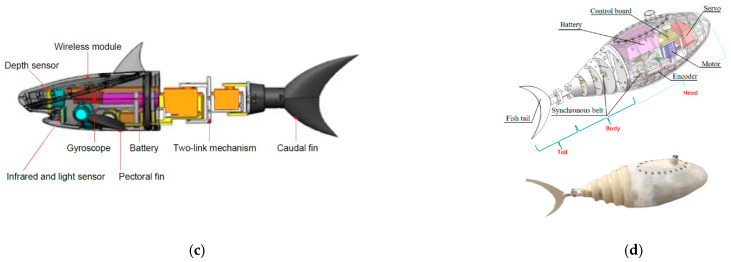
(**a**) Overview of the soft robotic fish design: schematic layout, side and top views with key dimensions, and terminology related to fish stability (Reproduced with permission from [155]); (**b**) Bioinspired robotic prototype designed with a streamlined morphology to reduce hydrodynamic drag. The schematic highlights internal architecture, including a brushless DC motor with an integrated encoder, which actuates a flexible tail section. Control and communication electronics are housed within the head module (Reproduced with permission from [43]); (**c**) Miniaturized robotic fish with compact mechanical architecture (Reproduced with permission from [156]); (**d**) Design and prototype of the robotic fish: schematic overview (Reproduced with permission from [157]).

These robotic fishes or flexible-fin AUVs often exhibit higher propulsion efficiency at moderate speeds than their propeller-driven counterparts, although top speeds may lag behind advanced marine propellers. However, the trade-off in agility can be advantageous for tasks such as reef exploration, pipeline inspection, or stealth operations. Various robotic fish designs have been developed to emulate the efficient swimming patterns of aquatic species. Examples include soft-bodied systems like soft robotic fish, which integrates sensing and buoyancy control within a flexible frame, as well as multi-joint robotic models featuring compliant structures for enhanced maneuverability (refer to Figure 16 [43,155,156,157]).

#### 5.1.2. Marine Mammals: Whales, Dolphins, and the Humpback Whale Phenomenon

Marine mammals, particularly whales and dolphins, offer another perspective on morphing. Their flukes (tail fins) operate in vertical oscillations, while pectoral flippers provide roll and pitch control. The humpback whale is famous for its “tubercled” flippers, whose leading-edge bumps can delay stalling and improve maneuverability. Dolphin and whale flukes are not just rigid slabs; they can exhibit chordwise flexibility. This elasticity helps manage flow separation and harness vortex structures for thrust generation. Some engineering concepts adapt flexible fluke-like propellers to mitigate cavitation, the formation and collapse of vapor bubbles that can damage conventional marine propellers and inhibit efficiency. A flexible fluke-inspired propeller can adapt pitch or camber to maintain a more favorable angle of attack, mitigating cavitation. By actively tuning fluke curvature, the same propulsor can operate efficiently from low-speed maneuvers to higher-speed cruising [158].

##### Bioinspired Leading-Edge Tubercles

Inspired by the humpback whale’s pectoral flippers, engineers have tested leading-edge tubercles on hydrofoils, rudders, and wind turbine blades. The bumps create streamwise vortices that energize the boundary layer, delaying flow separation. Although tubercles might increase drag at specific angles of attack, overall performance benefits often accrue across a wider range of flow conditions. Tubercles function with minimal or no active adjustment, an example of morphological adaptation that is largely static. However, some researchers have proposed morphing tubercles that can grow or shrink (via inflatable structures or shape memory materials), potentially tuning the vortex patterns in real time [159,160].

##### Implications for Large Vessels

Although whales employ tubercles at moderate scales, the same principle could be applied to large cargo ships or submarines, possibly at the segmented leading edge of a control surface. When combined with morphing capabilities, these tubercled edges can adjust the spacing, amplitude, or location of bumps to handle different speeds, loads, or sea states. Initial studies suggest benefits in yaw stability and energy efficiency, although scaling challenges and structural complexities remain interesting research topics [160].

#### 5.1.3. Cephalopods and Soft-Bodied Morphing

Cephalopods (octopuses, squids, and cuttlefish) are expert contortionists in marine environments. Although most of their locomotion depends on jet propulsion, several cephalopod species also have fins or webbing that they actively manipulate.

Certain squids have lateral fins that wave or undulate, adjusting their shape and amplitude for fine control, station-keeping, or reversing. Engineers replicate this with soft robotic fins that utilize fluidic actuators in a flexible membrane [41,161]. Some species alter their body volume, which can minimize or increase drag. Although not always directly relevant to typical underwater vehicles, this principle has inspired variable buoyancy systems that modulate hull volume or shape for stealth or energy-saving buoyancy control. Though primarily for camouflage, cephalopod skin possesses distributed pigment cells (chromatophores) and textural papillae that can alter surface roughness. Engineers envision underwater drones with adaptive skins that regulate boundary-layer characteristics or minimize detection by sonar. Such a “camouflage morphing” could be multi-functional, simultaneously modulating flow and reflectivity [162].

In general, cephalopods exemplify the soft robotics approach—large, continuous deformations with minimal rigid skeletons. While challenging to implement in high-speed contexts, these principles are valuable for slow-moving or highly flexible submersibles, particularly in cluttered or delicate environments (e.g., coral reefs and underwater archaeology sites).

#### 5.1.4. Leading- and Trailing-Edge Devices

Inspired by the flipper morphology of humpback whale flippers, morphing hydrofoils exhibit the potential to modify curvature or deploy small slots to manipulate boundary-layer flow. This adaptation facilitates the reduction of flow separation and the modulation of lift with minimal drag penalties. However, engineers face significant challenges regarding the waterproofing of actuators, ensuring structural integrity under high hydrostatic pressures, and mitigating biofouling (accumulation of marine organisms on surfaces).

#### 5.1.5. Passive Compliance for Flow Stabilization

An alternative to actively controlled morphing is passive compliance, where the foil’s material properties cause it to bend or twist under hydrodynamic loads in beneficial ways, without direct input from motors or SMAs. This approach can simplify design and reduce energy consumption. A prevalent example is observed in flexible fish caudal fins, which automatically adapt their shape to different speeds. By carefully tailoring its stiffness gradient, or embedding elastic rods, a hydrofoil can self-adjust underload, streamlining force spikes and diminishing drag [163].

### 5.2. Role of Morphing in Aquatic Environments

The ability to morph and adapt to changing environmental conditions is a defining characteristic of biological organisms in aquatic environments. Unlike rigid structures, morphing surfaces and adaptive hydrodynamic designs enable marine species to enhance their efficiency in propulsion, maneuverability, and stability.

**Figure 17:**
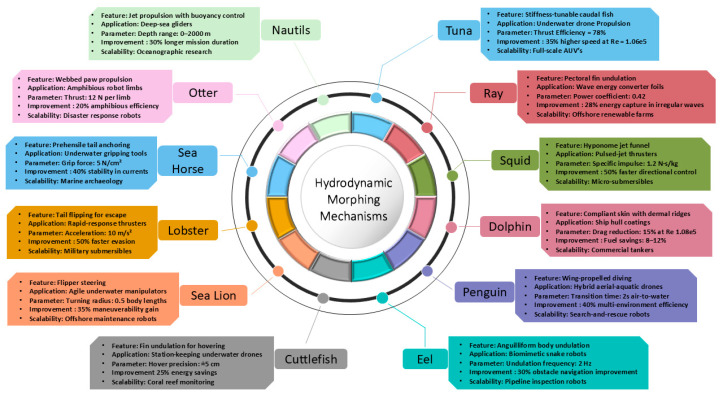
Bioinspired hydrodynamic morphing mechanisms across various aquatic species. Key features, applications, performance parameters, improvements, and scalability potential are indicated for each organism.

Engineers and researchers are increasingly exploring bioinspired morphing strategies in marine applications to improve performance, minimize energy consumption, and optimize interaction with unsteady flow conditions. Morphing in aquatic environments (refer to Aquatic Species—Figure 17) presents a unique set of challenges and opportunities compared to aerospace applications. The density and viscosity of water create significantly different flow conditions, making bio-inspired adaptation even more critical for reducing drag, increasing efficiency, and enabling multi-functional capabilities. This section explores the role of morphing in aquatic environments, considering its influence on hydrodynamic efficiency, propulsion, maneuverability, and energy harvesting.

#### 5.2.1. Hydrodynamic Efficiency and Drag Reduction

The primary role of morphing in aquatic environments is to reduce hydrodynamic drag and improve efficiency. Aquatic animals such as dolphins, sharks, and fish demonstrate remarkable adaptations that minimize resistance as they move through water.

**Figure 18:**
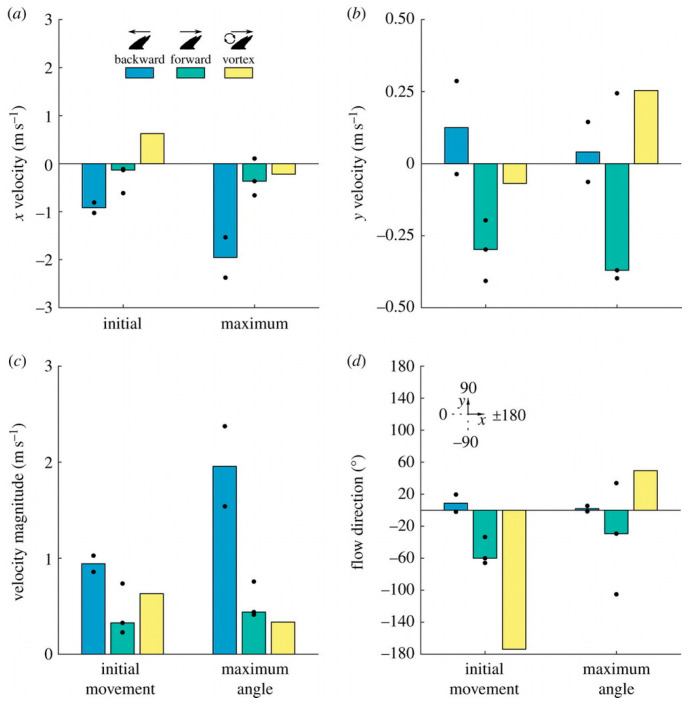
Flow velocity characteristics over shark scales were evaluated through particle tracking velocimetry (PTV) within a 0.2 × 0.2 mm region above the scale tip, capturing (**a**) median x- and (**b**) y-component velocities, (**c**) overall velocity magnitude, and (**d**) flow direction at both the onset of scale motion and peak scale extension. The analysis considered three distinct flow conditions: reverse jet (*n* = 2), forward jet over a barrier (*n* = 3), and forward jet with an upstream vortex (*n* = 1). Data points from individual video frames are represented as dots. Among the three, the reverse jet condition exhibited the highest flow velocities. Notably, initial scale activation in the forward jet case occurred without any observable reverse flow (Reproduced with permission from [165]).

*Sharks, for example, have flexible skin with riblet structures that passively modify turbulence and reduce drag. Shark skin features riblet structures aligned in the direction of flow, which can reduce skin friction drag in turbulent flow up to 10%. These riblets lift turbulent vortices away from the surface, minimizing shear stress and drag [164]. Shark scales and flexible skin contribute to drag reduction* via *passive flow control by managing vortex formation and improving boundary layer characteristics. This mechanism has inspired biomimetic designs for underwater vehicles and other engineering applications (refer to Figure 18) [165]. Similarly, dolphins use compliant, deformable skin to counteract flow instabilities and maintain smooth motion. Inspired by these natural adaptations, engineers have explored flexible hull coatings, compliant surfaces, and actively deforming bodies in underwater vehicles to optimize their performance.*

An approach to drag reduction is employing adaptive hull forms that adjust in response to water flow. Conventional ship hulls are optimized for fixed speeds, reducing efficiency in different ocean conditions. A morphing hull can adjust its curvature dynamically, adapting to different speeds or wave conditions to minimize drag and fuel consumption. Bioinspired flexible membranes, like ribbed surfaces, are utilized in AUVs to enhance performance by reducing boundary layer separation [166].

#### 5.2.2. Adaptive Propulsion for Enhanced Performance

Morphing plays a crucial role in propulsion efficiency, particularly in bioinspired swimming robots, AUVs, and marine turbines. Unlike conventional rigid propellers, biological swimmers rely on flexible and deformable appendages to achieve high propulsion efficiency with minimal energy loss. Fish fins, cetacean flukes, and squid-like jet propulsion systems utilize morphing kinematics to optimize thrust generation dynamically.

*For example, tuna and mackerel utilize high-aspect-ratio caudal fins that adjust their stiffness and shape during swimming, enabling precise control over thrust and maneuverability. Humpback whales have tubercle-textured flippers that dynamically modify lift and delay stall during rapid turns. These principles have inspired biomimetic underwater propulsion systems such as flexible propulsors, oscillating foils, and undulating robotic fish that outperform conventional rotary propulsion in maneuverability and efficiency.*

Morphing-enabled propulsion mechanisms can also replace rigid marine propellers, which suffer from cavitation and energy losses at varying speeds. Smart fin propulsion enables vehicles to adjust their stroke amplitude, fin flexibility, or oscillation frequency based on actual environmental feedback, resulting in more energy-efficient and quieter propulsion, critical factors for stealthy underwater operations in military and research applications.

#### 5.2.3. Maneuverability and Stability in Unsteady Flows

Aquatic morphing improves propulsion, maneuverability, and stability by quickly adapting shape to counteract external disturbances in unsteady flows. Organisms often adapt their body shapes to stay stable in turbulent water.

*Octopuses and cuttlefish exhibit extreme morphing capabilities, allowing them to change body shape and squeeze through tight spaces or generate thrust in multiple directions. Their soft, compliant structures offer insights into the design of soft-bodied underwater robots that can morph to avoid obstacles, maneuver through confined spaces, or maintain stability in strong currents [167].*

Another example is the bioinspired adoption of trailing-edge control in aquatic applications.

*Many fish adjust their tail fin curvature asymmetrically to execute rapid turns or maintain balance during slow swimming. This concept has led to the development of flexible rudders and adaptive stabilizers for submarines and AUVs, where morphing elements allow precise trajectory corrections without requiring excessive control effort [168,169].*

In addition, morphing can help marine structures counteract wave forces and improve station-keeping capabilities. For example, floating offshore platforms or ocean energy devices can integrate shape-adaptive elements to adjust to wave-induced loads, minimizing wear and improving endurance.

### 5.3. Bioinspired Hydrodynamic Energy Harvesting

A particularly promising domain for the application of bio-inspired morphing in hydrodynamics is renewable energy, wherein wave, tidal, and current energy converters are designed to exploit the substantial power resources of oceans and rivers. Conventional turbine designs often face performance and durability issues under variable flow conditions. By adopting morphing strategies akin to aquatic organisms, these devices can adapt their shape to optimize energy capture and minimize structural stress.

Tidal turbines function similarly to wind turbines but in denser water flows. They operate in highly dynamic environments, where flow velocity and direction can shift significantly. Fish-inspired morphing can be improved by adjusting pitch, camber, or even chord length in real time, such that the turbine can maintain an optimal angle of attack across varying tidal speeds. Harsh flow conditions can cause fatigue. Adaptive or compliant blades can flex under extreme loads, preventing catastrophic failures or excessive vibration. Some designs employ flexible joints, mimicking fish neck or shoulder joints, to orient the entire blade assembly into the flow with minimal mechanical complexity [170].

**Figure 19:**
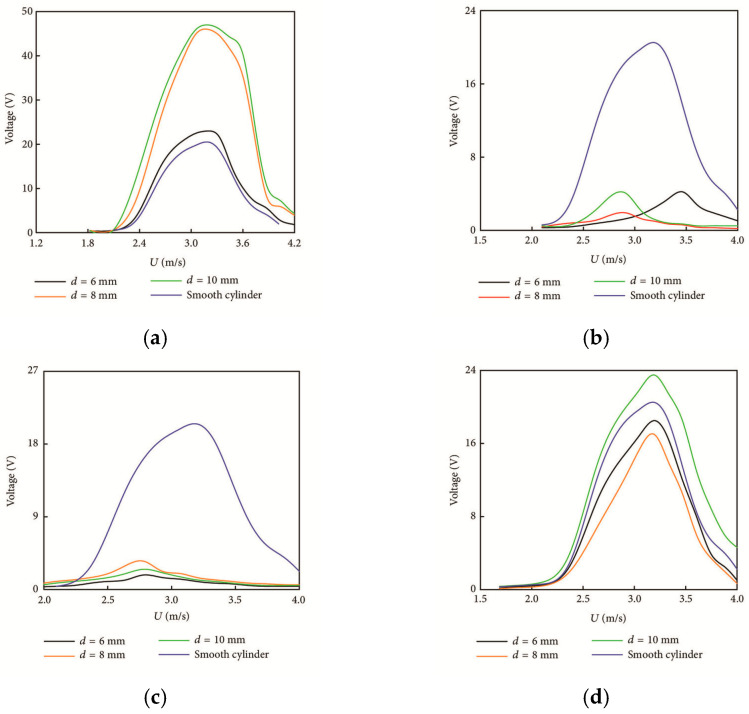
Vortex-induced vibration characteristics and output voltage response of convex bionic structures with varying hemispherical configurations. (**a**) Output voltage comparison for three-column hemispherical bionic structures with diameters of 6 mm, 8 mm, and 10 mm, indicating that the 10 mm configuration yields the highest voltage output (~50 V), representing a 100% enhancement compared to a smooth cylinder; (**b**,**c**) Output voltages for four-column and five-column configurations, respectively, showing significant suppression of vibration compared to the smooth cylinder, suggesting their potential for vibration mitigation in structural applications; (**d**) Voltage response versus wind speed for a six-column hemispherical bionic cylinder with varying diameters, demonstrating a non-monotonic trend where voltage initially increases and then decreases with increasing wind speed. The 10 mm hemisphere enhances vibration-induced energy harvesting, while 6–8 mm hemispheres act to suppress bluff body oscillation (Reproduced with permission from [173]).

Instead of rotating blades, some energy devices rely on oscillating foils that mimic fish or whale flukes. A foil is attached to a pivot, causing it to heave or pitch with the incident flow. By capturing the energy of this forced oscillation and converting it to electrical power (via mechanical linkages or direct electromagnetic couplings), these devices can achieve efficiency comparable to turbines, especially in moderate flow conditions [171,172]. Passive flapping harvesters simply respond to flow, while active systems incorporate sensors and actuators that tune the flapping frequency, amplitude, or foil shape for resonance and maximum power extraction, similar to how a fish might modulate its tail to match flow eddies. Observations of trout or eels exploiting vortex streets behind obstacles have led to “vortex-induced vibration” or “vortex-induced motion” devices. Through controlled morphing, these surfaces can further enhance vibrations, leading to increased power output and a widened operational range of flow speeds (refer to Figure 19) [173,174].

WECs, including point absorbers, oscillating water columns, and attenuators, rely on the interaction between wave motion and device structure. By dynamically altering buoy shape, a device can better match the incoming wave profile, extracting more energy per cycle. Some WEC concepts adopt large membranes that undulate with incoming waves. Morphing the membrane’s tension or curvature can align with wave crests and troughs more effectively, maximizing energy capture [175]. Creatures like sea lions or seals navigate wave-infested waters by adjusting their body posture. Translating these adaptive postures into wave-exploiting motions is a growing field, as the ocean environment is inherently complex.

### 5.4. Materials, Actuators, and Control for Underwater Morphing

Bioinspired morphing in water shares many challenges with aerodynamic morphing, including the selection of lightweight, high-strength, and flexible materials; the implementation of robust actuation systems; and the development of advanced control architectures. Nevertheless, it also encounters additional constraints like corrosion, hydrostatic pressure, and biofouling.

Stainless steel, titanium [176], or fiber-reinforced polymers are common for marine structures. Embedding or bonding smart materials (SMAs, piezoelectrics) requires careful encapsulation to protect against saltwater corrosion and infiltration [177]. For soft robotic or flexible fin designs, elastomers that maintain elasticity in cold saltwater are paramount. Some hydrogels can change volume or stiffness in response to ionic changes in water, hinting at the potential for environmentally driven morphing. Marine organisms (barnacles, algae) readily attach to surfaces, influencing morphing surfaces by adding weight and drag or jamming moving parts. Advanced coatings or self-cleaning surfaces can mitigate fouling, drawing inspiration from shark skin microstructures or other natural antifouling strategies [178,179]. Common in subsea operations, these fluid lines can be sealed; however, they are complex and heavy. Certain EAPs (IPMCs) can function directly in water, although controlling ionic balance and preventing electrode degradation is complicated [180]. Adjusting the viscosity of internal fluids under a magnetic field can help modulate stiffness or damping in morphing structures, useful for passive-active hybrids. SMAs can be enclosed in watertight housings or polymer coatings. Cooling in water is faster than in air, which can be an advantage; however, cyclical heating–cooling still requires robust thermal management [176,180].

Table 4 contrasts SMAs, PZTs, and EAPs by actuation mechanism, performance, cost, and typical uses. SMAs give large, high-force shape changes but are slower and heat-dependent. PZTs deliver rapid, precise motion with high force density yet only small strain, fitting high-frequency control tasks. EAPs supply large, compliant strain for soft robots and artificial muscles but face stability and high-voltage challenges.

Real-time control is crucial for dynamic morphing in swirling currents or wave-laden environments. Arrays of pressure sensors distributed on a morphing fin can detect local flow separation or vortex formation, prompting shape adjustments, which mirror fish lateral line sensing. Monitoring structural loads ensures that shape changes do not exceed safe stress levels and can inform closed-loop shape adaptation to reduce peak stresses. AUVs or underwater drones rely on IMUs to track orientation and acceleration. Combining IMU data with flow sensors allows sophisticated 6DOF motion control. Because wave or current conditions fluctuate unpredictably, ML-based controllers can adapt faster than human operators or fixed control laws, optimizing foil or fin positions in real time for better stability or power capture [179].

## 6. Research Methodology for Bioinspired Morphing Systems

Bioinspired morphing, whether in aerodynamics or hydrodynamics, relies on certain research techniques. These range from conventional theoretical models in fluid dynamics to advanced numerical simulations coupling FSIs to rigorous experimental validation in wind tunnels or water tanks, and finally to cutting-edge artificial intelligence (AI) and deep learning frameworks that optimize morphing performance. This section comprehensively reviews these methodologies, highlighting their relevance, challenges, and synergy in pushing the boundaries of adaptive, nature-inspired designs.

### 6.1. Theoretical Models

#### 6.1.1. Conventional Fluid Dynamics and Structural Models

Conventional fluid dynamics and structural mechanics form the foundation of research on bioinspired morphing. Before sophisticated computational methods became prevalent, researchers relied primarily on analytical models, approximations, and simplified assumptions to capture essential physics. For aerodynamics, one of the seminal approaches applies the potential flow theory, particularly for inviscid, incompressible flows [181]. While potential flow ignores viscous effects, it provides useful insights into pressure distribution and lift generation over an airfoil. Early morphing-wing studies often began by examining potential flow solutions around streamlined bodies that could change camber or thickness.

Simultaneously, structural models were derived from the beam theory and plate/shell theory. Small deflections in a wing or fin could often be approximated using conventional linear models [182]. However, bioinspired morphing typically requires large deflections, nonlinear material behavior, and variable geometry. Conventional linear elasticity is limited; to replicate the large, dynamic shape changes observed in nature (e.g., a bird’s wing morphing from high lift take off to efficient cruising), engineers or biologists had to consider geometric and material nonlinearities [183].

In hydrodynamics, theoretical modelling draws upon unsteady flow theories such as Theodorsen’s theory, historically used to oscillating airfoils, or Lighthill’s elongated-body theory for fish-like swimming. These methods attempt to capture the interplay between body deformation and fluid acceleration. For fish locomotion, the elongated-body theory postulates a distribution of vorticity along a flexible body. When the body flexes in a traveling wave, it moves the fluid backward to generate thrust. Though these models can be simplified, they are essential stepping-stones guiding researchers toward more nuanced investigations [50].

Classical models also rely on momentum and energy considerations. For example, analyzing the momentum flux in the wake of a morphing wing or fin can yield approximate thrust and efficiency measures [184]. While these simplified approaches sometimes overlook boundary-layer effects or three-dimensional complexities, they remain invaluable. In addition, they provide essential baseline understanding, help verify more advanced computational or experimental data, and offer quick initial assessments of design feasibility.

#### 6.1.2. Bioinspired Optimization Algorithms

Nature itself is a powerful optimizer, having honed the morphology of birds, fish, insects, and mammals over millions of years. To reflect this, engineers have developed bioinspired optimization algorithms, including genetic algorithms (GAs), particle swarm optimization (PSO), ant colony optimization, and differential evolution. These methods mimic the processes of natural selection, swarming, or foraging, enabling computational “populations” of candidate solutions to evolve over successive generations [185].

Regarding morphing structures, researchers might encode parameters like airfoil shape, fin kinematics, material thickness distributions, or actuator placement into a “genome.”

A function—such as lift-to-drag ratio, propulsive efficiency, maneuverability, or even noise reduction—guides the evolutionary process. Over multiple generations, these algorithms systematically “breed” new shapes or kinematic patterns from the fittest designs.

A notable advantage is robustness in complex design spaces.

*Actual morphing systems can exhibit multiple local minima, strong parameter interactions, and complex fluid–structure couplings. Conventional gradient-based optimizers often get stuck in local optima, while bioinspired methods are more adept at global exploration. They can also incorporate multi-objective criteria, for instance, maximizing lift while minimizing power consumption. By analyzing the Pareto front of solutions, engineers can observe trade-offs between competing objectives [186,187].*

Nevertheless, using bioinspired algorithms for morphing design can be computationally intensive, especially when each candidate solution requires a high-fidelity fluid–structure simulation. Researchers often integrate these algorithms with surrogate models or reduced-order models (ROMs) to minimize runtime. Surrogate models approximate the system’s behavior based on a limited set of high-fidelity data points, thus speeding up subsequent evaluations. Despite computational challenges, bioinspired optimization is a fundamental methodology for the systematic exploration of expansive design spaces in pursuit of novel, high-performance morphing concepts [188,189].

#### 6.1.3. Multi-Scale Modelling for Complex Morphing Mechanisms

Bio-inspired morphing can encompass phenomena at multiple scales, ranging from microscopic material behavior in smart materials (e.g., shape-memory alloys) to macro-level structural deformations in an entire aircraft wing or submarine hull. Hence, multi-scale modelling is crucial. It involves coupling continuum mechanics at the macroscale with microscopic or mesoscopic models that capture the physics of active materials, such as piezoelectric elements, EAPs, or magneto-rheological fluids [181].

Homogenization is an approach where the detailed features of a material’s internal structure are averaged to obtain effective macro-scale properties. For instance, if a morphing wing incorporates an internal lattice of piezoelectric actuators, each element may exhibit complex local behavior; in contrast, a homogenized model yields “bulk” properties that can be implemented in a structural finite element (FE) framework [190]. Conversely, XFEM (extended FE method) or advanced mesh-free methods can manage discontinuities or large deformations that emerge when integrating flexible, deployable, or inflating structures [191].

Multi-scale modelling also extends to fluid mechanics. A fish’s scale or a humpback whale tubercle might exhibit micro-vortex generation, influencing the boundary layer to minimize drag. The accurate capture of these subtle microscale phenomena can, consequently, induce alterations in macroscale flow patterns [192]. Integrating such details into a single framework requires advanced computational resources, partitioned solvers, and well-designed bridging strategies. Multi-scale modelling stands at the frontier of understanding and predicting performance in complex bio-inspired morphing systems, ensuring that results considered at one scale do not overlook critical phenomena at another.

### 6.2. Computational Approaches

#### 6.2.1. CFD for Morphing Aerodynamics and Hydrodynamics

CFD has become essential for analyzing and designing morphing systems. By numerically solving the Navier–Stokes equations (or simplified forms in certain regimes), researchers can predict pressure, velocity, temperature, and vorticity fields around a morphing structure [193]. Regarding aerodynamic studies, CFD can help optimize wing camber, leading-edge devices, or winglets to maintain high lift-to-drag ratios under changing flight conditions. For hydrodynamic applications, CFD reveals how the changing shape of a fin or hull modifies wake patterns and thrust generation [194].

A major challenge is that morphing surfaces introduce time-varying geometry. Conventional CFD utilizes static geometry; hence, morphing requires dynamic meshing (adjusting the mesh at each time step) or immersed boundary methods (embedding the morphing body in a fixed mesh). Dynamic remeshing can be computationally expensive, especially in 3D problems with fine boundary layers. Although immersed boundary methods circumvent some meshing complexities, they can introduce interpolation errors near the fluid–structure interface [194].

**Figure 20:**
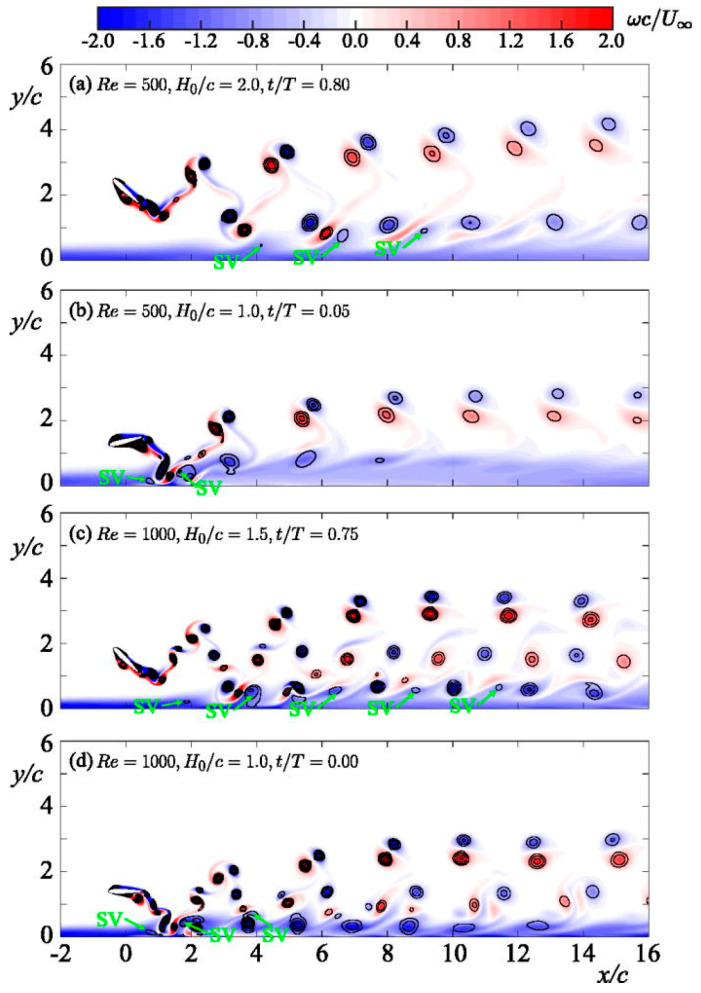
Flow vorticity patterns near a flapping wing adjacent to a wall. This figure presents normalized vorticity distributions (ωc/U_∞_) and λ_2_ vortex identification contours for a flapping wing under the following conditions: (**a**) Re = 500, H_0_/c = 2.0, t/T = 0.80; (**b**) Re = 500, H_0_/c = 1.0, t/T = 0.05; (**c**) Re = 1000 Re = 1000 Re = 1000, H_0_/c = 1.5, t/T = 0.75; (**d**) Re = 1000, H_0_/c = 1.0, t/T = 0.00. Secondary vortices (SV) are marked with green arrows, emphasizing their development near the boundary layer at a specific moment (Reproduced with permission from [195]).

In unsteady morphing cases like flapping wings or dynamic shape adaptation in response to turbulence, time-accurate simulations (e.g., large eddy simulations (LES) or direct numerical simulations (DNS)) may be required. LES and DNS can capture the intricate vortex structures and turbulent eddies that develop around flexible bodies, as illustrated in Figure 20 [195]. However, these methods require substantial computational power, making them feasible mostly for smaller Reynolds numbers or simplified geometries. Although Reynolds-averaged Navier–Stokes (RANS) remains the conventional approach for large-scale engineering design owing to its lower computational cost, it might not comprehensively resolve the rich unsteady flows that morphing systems exploit [196].

To advance bio-inspired morphing systems from concept to reliable design, both the underlying structures and the supporting CFD tools must be systematically upgraded. Recent breakthroughs in hyper-elastic skins and megastructures with tunable stiffness provide new avenues for dynamic shape control [197], while multi-objective optimization of flapping-wing kinematics [198] and genetic-algorithm tuning of Voronoi-tessellated cores [199] illustrate the power of computational design. At the simulation level, accuracy and efficiency are boosted by (i) adaptive or overset meshes that capture complex, time-varying geometries without excessive cells; (ii) a turbulence-model hierarchy—RANS for rapid screening, LES or DNS for high-fidelity unsteady flow; (iii) robust partitioned or monolithic FSI coupling to resolve two-way fluid–structure interactions; (iv) rigorous validation against PIV and force data; and (v) data-driven surrogates or neural-network regressors that speed optimization and enable near-real-time predictions. Together, these structural innovations and CFD enhancements transform morphing research from descriptive analysis into a predictive, design-optimization framework.

Despite these challenges, CFD remains indispensable. It (1) accelerates design iterations, (2) offers detailed flow field data not always accessible via experiments, and (3) guides parametric studies. Coupled with optimization algorithms, CFD can automate the search for an ideal morphing schedule or geometry. As computing resources continue to scale, and as GPU-accelerated or high-performance computing clusters become more accessible, CFD-based analyses of morphing systems are expected to grow even more prominent [200].

#### 6.2.2. FSI Simulations for Adaptive Morphing

Morphing often involves significant structural deformations that, in turn, alter the fluid flow, an interplay already introduced as FSI. FSI models consider the fluid domain and structural domain as coupled, exchanging pressure, shear stresses, and displacement at the interface. For example, a flexible fish fin or adaptive wing experiences fluid forces that bend or twist the structure, which then changes the flow, forming a feedback loop.

FSI can be approached using either monolithic or partitioned methods. Monolithic solvers embed both fluid and structural equations in a single system, simultaneously solving fluid flow and structural displacement. Although this approach is often robust, it can be computationally limited for complex 3D problems. Conversely, partitioned solvers use separate, specialized codes for the fluid and the structure, exchanging data at each time step via a coupling interface. This can be more flexible but is sometimes inhibited by numerical instabilities, especially in highly coupled or high-density ratio problems like water-submerged structures [201].

A classic example is the study of flapping hydrodynamic propulsors.

*Using FSI, one can capture the large amplitude, nonlinear deformation of fins, including the wake vortex structures that contribute to propulsion. In aerodynamics, morphing winglets or variable-camber wings can be optimized with FSI to ensure that structural deflections remain within safe limits while maximizing aerodynamic efficiency [202,203].*

Key challenges include mesh handling, time stepping, and ensuring numerical stability when handling large-scale deformations. In addition, the material models in the structural domain can be highly nonlinear, such as hyper-elastic membranes used in bat-inspired wings or shape-memory alloys with thermo-mechanical coupling. Each new complexity adds to the computational load and the intricacy of solver coupling.

Recently, high-fidelity FSI codes have started integrating advanced turbulence models (like LES or hybrid RANS–LES) on the fluid side and elasto-plastic or viscoelastic models on the structural side. This synergy is particularly relevant for bio-inspired morphing, where natural tissues and engineered composites can exhibit viscoelastic or anisotropic properties. FSI simulations, while demanding, deliver crucial insights into how morphing performance emerges from the dynamic interplay of structure and fluid [204].

#### 6.2.3. Vortex Dynamics and Flow Control Simulations

Natural flyers and swimmers primarily morph to generate, control, or exploit vortices. Birds, bats, and insects rely on LEVs for lift augmentation [205]. Fish and dolphins harness vortex shedding for efficient propulsion. Vortex dynamics simulations attempt to capture these phenomena comprehensively. Techniques vary from vortex-lattice methods, commonly used in preliminary aerodynamic analyses, to discrete vortex methods for unsteady flows around flapping or rotating bodies [206].

Flow control can be active (e.g., using actuators on the surface to control boundary-layer separation) or passive (e.g., tubercles on whale flippers to delay stall) [192]. Morphing surfaces combine passive elements, such as a leading-edge flap aligning with flow, and active elements controlled by embedded actuators. Simulation frameworks that emphasize vortex evolution can highlight how minor changes in geometry significantly influence vortex shedding frequency, strength, and stability [207].

Researchers sometimes couple these vortex-based methods with nonlinear control strategies. For instance, an embedded sensor can measure local pressure fluctuations, detect an impending stall vortex, and trigger a shape change to reattach the flow [208]. The simulation domain must therefore incorporate control laws that respond to actual flow conditions. While more specialized than standard CFD, vortex-based simulations can be more computationally efficient and could provide more significant insight into unsteady fluid phenomena crucial to morphing.

#### 6.2.4. Bioinspired Optimization Method

Bio-mimetic optimization techniques translate evolutionary principles into efficient design search engines for morphing structures. Genetic Algorithms (GAs) emulate natural selection to sift through large, multi-dimensional design spaces and pinpoint wing- or fin-shapes that maximize lift-to-drag or thrust-to-power ratios [197]. Particle Swarm Optimization (PSO) harnesses swarm-intelligence heuristics to refine coupled variables such as local material stiffness and hinge locations, converging rapidly on high-performance solutions [209]. When these heuristic methods are paired with machine-learning surrogates—deep neural networks or Gaussian-process regressors trained on CFD/FSI data—the optimization loop accelerates by orders of magnitude, enabling real-time re-shaping or on-the-fly sensitivity studies [210]. Collectively, these algorithms allow engineers to explore complex, non-intuitive parameter combinations and deliver morphing devices that achieve superior aerodynamic or hydrodynamic efficiency, agility, and robustness compared with traditional design approaches.

### 6.3. Experimental Approaches

#### 6.3.1. Wind Tunnel and Water Tank Testing of Morphing Structures

Despite advances in computational modelling, experimental validation remains essential. Wind tunnel tests in aerodynamics and water tank (or flume) tests in hydrodynamics provide controlled environments to measure forces, moments, pressure distributions, wake velocity fields, etc. [211]. The complexity of actual flow conditions—boundary-layer transitions, turbulence, and material imperfections—can challenge purely numerical predictions. Hence, experiments function as a reality check, informing and sometimes correcting computational assumptions.

**Figure 21:**
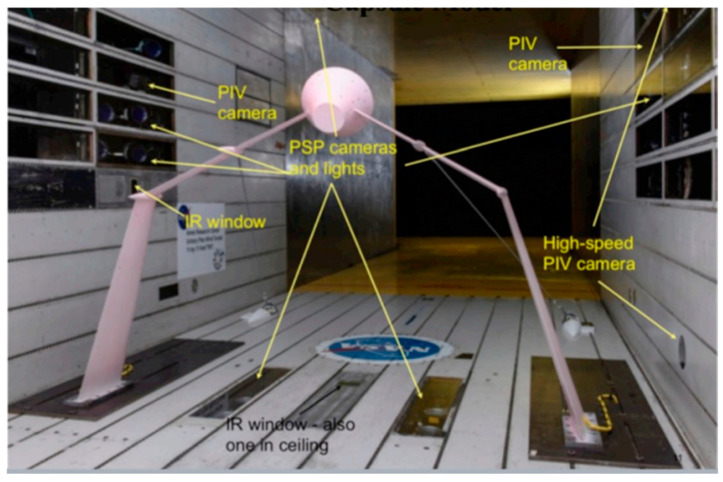
Public-domain photograph of the 11 × 11 ft unitary plan wind tunnel (nasa ames) showing a fully instrumented generic-capsule model. Optical windows on the test-section walls enable simultaneous particle image velocimetry and pressure-sensitive paint measurements, while internal pressure taps and an external six-component balance record forces and moments. The setup exemplifies the synchronized, multi-sensor approach required to validate morphing-wing aerodynamics under realistic Reynolds numbers. Reproduced from Roozeboom and Baerny (2019), NASA/TM-2019-27089, Figure 3 [212].

Scaled models of morphing wings or fins are often assessed, ensuring similarity in Reynolds numbers where possible. However, owing to the difficulty of exact dynamic similarity in unsteady conditions, Reynolds number mismatch may remain. Researchers employ advanced measurement techniques like particle image velocimetry to visualize the flow field and pressure-sensitive paint, or pressure taps to obtain local pressure data (Figure 21 [212]). High-speed cameras can capture structural deformation in real time, enabling correlation with flow phenomena [213].

One challenge is that morphing typically involves time-varying geometry. Gimbals, servo motors, or other mechanical linkages might be used in wind tunnel setups to replicate dynamic shape changes. The instrumentation and synchronization required can get complicated. In water tanks, flexible robotic fins or self-propelled fish-like robots are evaluated at varying speeds or wave conditions, analyzing how changes in fin stiffness or shape influence propulsion efficiency [214]. The data obtained from these setups can reveal intricate vortex patterns, leading-edge separation, or boundary-layer states that purely numerical models might miss or oversimplify.

#### 6.3.2. Bioinspired Wing and Fin Prototyping

Several laboratories worldwide construct full or near-full-scale prototypes of bio-inspired morphing wings, fins, or entire vehicles, such as an MAV with a flapping membrane wing [215]. Prototyping is crucial for exploring material feasibility, actuator response, energy consumption, and practical constraints like size, mass, and cost. For aerodynamic prototypes, these might incorporate flexible skins made of advanced composites, shape-memory alloy rods for actuation, or pneumatic networks that inflate or deflate to alter geometry [216]. In hydrodynamic prototypes, researchers often mimic a fish’s segmented backbone or a dolphin’s fluke’s tendon-driven structure, using servo motors or cable-pulley systems to replicate bending waves [217]. Soft robotics approaches have also garnered attention, especially in cephalopod-inspired systems [218], where shape change is more continuous and less reliant on rigid internal frames.

*A critical advantage of prototyping is the ability to measure real performance metrics: thrust, efficiency, maneuverability, or stall onset, which can be measured directly. Coupled with motion tracking and flow visualization, prototypes generate invaluable data for validating multi-physics models. This iterative loop—modelling → prototyping → testing → refinement—fuels the discovery of novel morphing concepts not easily predicted by theory alone.*

#### 6.3.3. Soft Robotics and Smart Materials Testing

Bioinspired morphing often involves soft materials that replicate the compliance and adaptability of biological tissues. Soft robotics research, using elastomers, hydrogels, and pneumatic artificial muscles, demonstrates how shape alterations can be more fluid-like and less reliant on bulky mechanical linkages [219]. For instance, an octopus-inspired arm might rely on an internal arrangement of pneumatic chambers. When inflated or deflated in a controlled pattern, the arm bends and twists in a continuous, organic manner [220].

Evaluating such soft-robotic morphing structures involves specialized characterization methods. Researchers measure stress–strain curves, hysteresis behavior, actuation speeds, energy consumption, and failure modes. Submerging them in a flume or attaching them to a robotic fish body can illustrate how well they produce propulsion or maneuvering forces [42]. Because soft materials can degrade, absorb water, or exhibit large hysteresis, long-term durability tests are required. In addition, compatibility with sensors (e.g., strain gauges, embedded optical fibers) is essential for real-time feedback control [221].

For smart materials (e.g., piezoelectric composites, SMAs, or EAPs), typical experiments measure actuation strain, block force, and frequency response [222]. By systematically varying temperature, voltage, or electric fields, researchers can determine optimal operating conditions. Incorporating these in morphing prototypes, whether aerial or aquatic, reveals real-world feasibility and clarifies the trade-offs between actuation speed, power consumption, and structural strength.

### 6.4. AI and Deep Learning AI-Driven Design Optimization for Morphing Structures

The complexity of bio-inspired morphing systems—featuring dynamic fluid-structure interactions, advanced materials, and wide design spaces—makes AI-driven design optimization particularly desirable. Building on the concept of bio-inspired optimization algorithms (like GAs), modern approaches leverage machine learning (ML) or deep neural networks to approximate high-dimensional design spaces. In several workflows, a neural network (NN) learns the mapping from design variables (e.g., wing geometry, actuation parameters) to performance metrics (e.g., lift, drag, efficiency).

A common strategy is to create a surrogate model: researchers run a series of high-fidelity simulations or experiments for distinctive design points, then train an NN or another regression model (e.g., Gaussian Processes, random forests) to approximate the function that links design inputs to performance outputs. Once trained, the surrogate can quickly evaluate modern designs, enabling faster iterative searches. Hybrid methods combine GAs or PSO with surrogate models to manage multi-objective problems, unveiling Pareto-optimal shapes or kinematics [223].

Another trend is utilizing autoencoders or deep generative models to represent complex shapes like flexible fins or morphing wings in a low-dimensional latent space [224]. Engineering designers can explore this latent space for feasible shape variations. The generative aspect ensures morphological continuity and physically realizable designs. Coupled with evolutionary search or RL, these methods can “discover” novel morphing solutions that might be overtly unintuitive for conventional parameterization strategies [225].

#### 6.4.1. Deep Learning-Based Flow Prediction and Control

Beyond design, deep learning has proven valuable for flow prediction and real-time control of morphing systems. Conventional CFD can be expensive if run online or in the loop of a control system. ROMs, such as proper orthogonal decomposition, have been popular for decades. Deep neural networks now predict time-evolving flow fields from limited sensor inputs. A well-trained neural network can approximate velocity or pressure distributions near a morphing surface with only partial data (e.g., from a few strategically placed pressure sensors) [226].

Researchers also explore RL for active flow control. In RL, an “agent” (the morphing system) receives a “reward” (improved lift, reduced drag, or minimized energy use) by adjusting its shape in real time [227]. Using numerous training episodes, often conducted in simulation, the agent develops a control policy that adjusts the geometry according to different flight or swimming conditions. This approach can replicate the adaptability seen in biological organisms, which continuously sense and respond to changing environmental flows.

In practice, RL-based control might be too computationally demanding to train purely high-fidelity CFD. Instead, researchers might rely on simplified or partial simulations or surrogate models for training and then apply the learned policy to real experiments [228]. As computational resources increase and domain randomization techniques improve, we can expect more robust and generalizable RL solutions for morphing wings, fins, and multi-limb propulsion systems.

#### 6.4.2. RL for Adaptive Morphing Systems

While flow prediction is about passive inference (the network predicts states), RL is active. For morphing systems, RL maps sensor readings (e.g., local flow velocity, pressure, and strain) to an actuation command that alters the structure (camber, deflection, and twist) to optimize a performance metric [225]. This metric could be energy efficiency in a UAV or maneuverability in an underwater robot.

A key challenge is sample efficiency. RL typically requires thousands or even millions of “episodes” to learn an optimal policy. Running a high-fidelity FSI simulation for such an extended time is impractical. Researchers sometimes employ multi-fidelity approaches: rapid training in a simplified environment or reduced-order model, followed by fine-tuning in smaller sets of high-fidelity simulations or real-world experiments [229]. Another approach is transferring learning, where an RL policy learned in a simplified domain is adapted to a more complex domain, such as the conversion of a 2D flow problem to a 3D representation requiring retraining [230]. With advancements in physics-informed neural networks and domain randomization, RL-based morphing control might become more robust to uncertainties like sensor noise, unpredictable turbulence, or hardware imperfections [231]. This yields a morphing system that adapts for peak efficiency or agility under varying conditions, similar to living organisms.

The workflow adopted in this review begins with conventional potential-flow analysis, progresses through high-fidelity FSI, and culminates in AI-driven optimization, as summarized in Table 5.

### 6.5. Integrating Multi-Disciplinary Approaches

#### Combining CFD, Experiments, and AI for Holistic Morphing Studies

**Figure 22:**
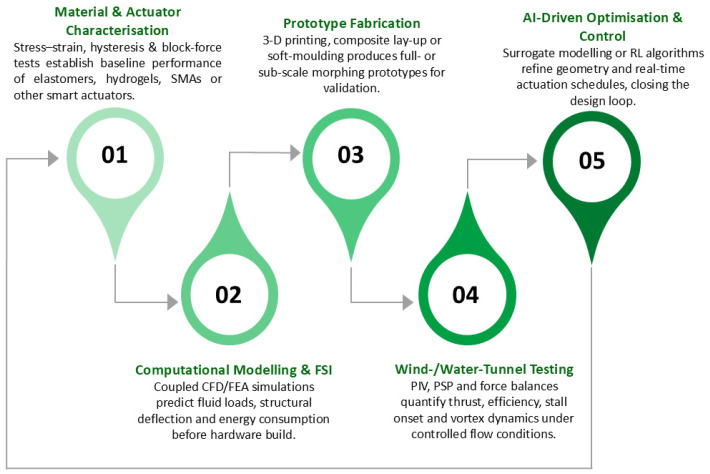
Iterative Development Cycle for Bioinspired Morphing Systems. The pipeline begins with material and actuator characterization (Step 1), proceeds to coupled computational modelling (Step 2), and then to full- or sub-scale prototype fabrication (Step 3). Laboratory wind-tunnel or water-flume tests provide performance data (Step 4) that feed AI-assisted optimization and control refinement (Step 5), closing the gap between simulation and experiment.

Due to the complex interactions inherent in bioinspired morphing, a comprehensive understanding necessitates a multi-pronged approach, as pure theory, stand-alone CFD, or isolated experiments are each insufficient to fully capture the interplay of structural deformation, flow physics, and control. Consequently, a common research paradigm is “CFD–Experiment–AI Triangulation.” Here, high-fidelity CFD can be employed to assess a range of design or control parameters, collect experimental data to validate and correct assumptions, and then employ AI to optimize or predict performance within that validated design space. Figure 22 presents the iterative development cycle, illustrating how experiments, simulations, and AI methods feed back into each other.

This cyclical process facilitates cross-pollination. Experimental data corrects or calibrates CFD, and CFD provides comprehensive flow fields that are complex to measure experimentally. AI reduces the computational burden of repeated design optimization or real-time control, harnessing knowledge derived from numerical and physical domains.

## 7. Engineering Challenges and Future Directions

Bioinspired morphing in aerodynamics and hydrodynamics can potentially transform how engineered systems interact with fluid flows. Designers replicate nature’s adaptive strategies to develop aircraft, underwater vehicles, and energy-harvesting devices that modify their shapes for better efficiency, stability, and functionality. However, the practical implementation of these concepts necessitates overcoming numerous engineering challenges spanning materials science, structural design, actuation, control, manufacturing, economics, and regulatory frameworks. This section provides a comprehensive overview of these challenges and explores future directions that could guide the continued evolution and adoption of morphing technologies for aerospace, marine, and renewable energy applications.

### 7.1. Material and Structural Challenges

Bioinspired morphing systems require advanced materials and structural configurations that can accommodate significant deformations while maintaining reliable performance over extended lifetimes. Designing and fabricating these systems introduces several challenges, primarily related to durability, mechanical trade-offs, and environmental exposure. Addressing these issues is key to transitioning morphing technologies from research prototypes to real-world applications in aeronautics, marine engineering, etc.

#### 7.1.1. Durability of Morphing Components

What and Why: Morphing skins must survive thousands of large-strain cycles without cracking or delaminating. Achieving a light structure that still offers high fatigue life is the central material science challenge. Morphing skins must resist microcracking and delamination under cyclic loads (refer to Table 6).

Research Requirements:

- Quantify multi-axial fatigue life in saline and UV-rich environments.
- Embed fiber-optic sensors with <5% mass penalty.
- Develop predictive life-cycle models coupling creep and micro-cracking.

#### 7.1.2. Trade-Offs Between Flexibility and Strength

What and Why: Although high flexibility reduces actuation power, it can compromise load capacity; conversely, excessive stiffness inhibits shape modifications. Balancing these competing demands requires targeted material and structural designs. Table 7 summarizes the core flexibility-rigidity tension and its resolution via hybrid laminates.

Research Requirements:

- Create fast FEA-based surrogates for early-stage design.
- Validate graded-laminate concepts under operational loads.
- Integrate topology optimization with manufacturing constraints.

#### 7.1.3. Environmental Exposure and Degradation

What and Why: Morphing systems are often exposed to UV radiation, saltwater, chemicals, and thermal cycles that accelerate aging and delamination. Robust environmental protection is vital for long-duration missions. Bioinspired morphing systems require UV-blocking and corrosion-resistant measures (refer to Table 8).

Research Requirements:

- Develop accelerated life-testing protocols for hybrid materials.
- Engineer microcapsule-based self-healing that activates in situ.
- Model coupled creep, swelling, and micro-delamination over time.

### 7.2. Manufacturing and Scalability Issues

Mainstream adoption of morphing hardware hinges on precise fabrication, cost management, and feasible scaling processes.

#### 7.2.1. Key Challenges and Short-Term Fixes

High-performance morphing hardware requires exotic materials, bespoke tooling, and extensive certification, factors that increase unit costs and require compelling lifecycle-return analyses before industries like aviation or maritime industries can commit to production. Table 9 pairs the principal barriers to scaling up from prototype to volume production (materials, tooling, certification, supply chain, and workforce) with interim strategies for risk and cost minimization.

#### 7.2.2. Scaling to Volume and Long-Term Vision

Even with reliable prototypes, scaling up to hundreds or thousands of morphing components exposes supply-chain fragilities, workforce skill gaps, and quality-control challenges that must be systematically addressed for mass manufacture.

- Automated Quality Assurance: Digital-twin process control, machine-vision inspection to maintain µm-scale alignment across hundreds of parts.
- Economies of Scale: Transition from custom prototypes to modular subassemblies and batch-fabrication to drive down per-unit costs.
- Ecosystem Partnerships: Co-funded manufacturing consortia (OEMs, regulators, materials suppliers) to share risk, standardize processes, and validate supply chains.

### 7.3. Actuation and Control Challenges

#### 7.3.1. Coupled Fluid-Structure Dynamics

What and Why: Morphing shapes and fluid loads interact bidirectionally, creating complex FSI behaviors (flutter, instabilities) that conventional aeroelastic methods cannot predict. Capturing vortex-morphing coupling requires co-simulation and ROMs (refer to Table 10).

Research Requirements:

- Develop physics-informed ROMs for real-time FSI prediction.
- Validate the-co-simulation strategies against PIV/strain-gauge experiments.
- Integrate passive morphological features into damp instabilities.
- Energy-Efficient Actuation Strategies

What and Why: Actuators are required to produce large deflections without compromising efficiency gains; smart materials and hybrid schemes are key to low-power morphing. Comparison of actuator types (SMAs, piezo, hydraulics) and energy-efficient approaches (bi-stable snap-through, hybrid multi-actuator schemes) (refer to Table 11).

Research Requirements:

- Quantify energy budgets for hybrid actuator systems.
- Develop bistable mechanisms with tunable snap thresholds.
- Embed energy-harvesting to offset sensor/control power.

#### 7.3.2. Real-Time Sensing and Control for Dynamic Morphing

What and Why: Millisecond-scale gusts or wave changes require distributed sensing and advanced control algorithms (INDI, MPC, RL) to optimize shape continuously. Real-time INDI/QP allocation and deep-RL for over-actuated wings are detailed in Table 12.

Research Requirements:

- Demonstrate INDI/MPC on hardware in the loop morphing rigs.
- Explore RL-based adaptive controllers with safety guarantees.
- Integrate fiber optic and MEMS sensors for sub-MS feedback loops.

Table 13 summarizes the principal engineering challenges (materials, manufacturing, actuation, and control), their biological inspirations, short-term mitigation strategies, long-term visions, and the key innovations required to close each gap.

## 8. Translational Value and Multi-Domain Impact of Bioinspired Morphing

Bioinspired morphing systems, rooted in the dynamic adaptability observed in natural organisms, are increasingly recognized for their aerodynamic and hydrodynamic advantages and broader economic, environmental, and societal implications. These adaptive technologies, once confined to laboratory exploration, are now making tangible impacts in multiple sectors ranging from aviation and healthcare to infrastructure and agriculture.

*For example, bioinspired morphing wings have demonstrated the ability to adapt their shape for optimal aerodynamic performance, resulting in improved maneuverability and efficiency in drones and aircraft, while similar principles are being applied in biomedical devices and robotics to enhance adaptability and functionality [134,253].*

**Figure 23:**
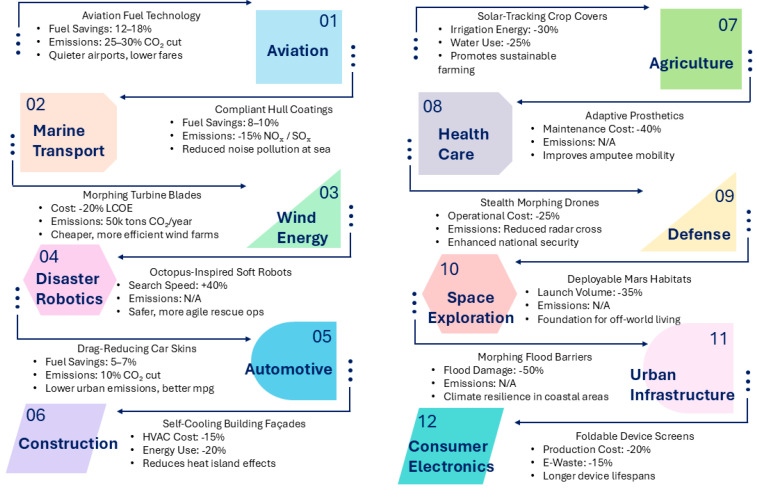
Economic and Environmental Impact.

Recent reviews and sectoral analyses highlight the adoption of morphing systems—such as variable-camber wings, shape-morphing hydrogels, and self-healing composite structures—to drive sustainability, resilience, and technological evolution [254,255]. Figure 23 summarizes these diverse applications, revealing how morphing systems can facilitate sustainability, resilience, and technological evolution. This section synthesizes these sectoral impacts, highlighting how morphing becomes a transformative force across real-world systems.

### 8.1. Aviation: Fuel Efficiency and Quieter Operation

Adaptive morphing surfaces in aviation, such as variable camber wings and seamless control surfaces, enhance aerodynamic efficiency by reducing drag and optimizing lift under varying flight conditions, which results in:

- Fuel savings of approximately 12–18% [256]
- Emission reductions of 25–30% [257]
- Lower noise levels during take-off and landing

In addition to minimizing operational costs, these improvements help aviation sectors satisfy stringent environmental regulations, making morphing designs key facilitators of next-generation sustainable air transport, thereby expanding the suitability of feasible renewable energy solutions.

### 8.2. Marine Transport: Adaptive Hulls for Energy and Noise Reduction

Inspired by the flexible bodies of marine animals, morphing hull coatings and compliant structural elements allow ships to minimize hydrodynamic resistance dynamically. These systems contribute to:

- Fuel savings of 8–10%
- Emission reduction of NO_x_/SO_x_ pollutants by up to 15%
- Minimized underwater noise pollution, benefiting marine life

This makes morphing technology valuable in both commercial and naval maritime operations, especially in ecologically sensitive or emission-regulated regions [258].

### 8.3. Wind Energy: Lower Costs and Higher Efficiency

Morphing wind turbine blades enable shape adaptation to varying wind speeds, maximizing energy capture while reducing mechanical stress. The impact includes:

- 20% reduction in the levelized cost of energy
- 50,000 tons/year CO_2_ reduction per large-scale installation
- Enhanced power output reliability under unsteady flow

These advantages position morphing turbines as economically attractive solutions for future offshore and onshore renewable energy deployments [259].

### 8.4. Disaster Robotics: Flexible Mobility in Hazardous Environments

Inspired by cephalopods and soft-bodied organisms, morphing robotic platforms for disaster response can squeeze through debris, adapt to unstable surfaces, and manipulate delicate objects. Key benefits include:

- 400% improvement in reach and maneuverability
- Emission-free operation
- Enhanced safety in high-risk rescue scenarios

In addition, these systems are critical in post-earthquake, fire, or flood environments where conventional robots may be limited [260,261,262].

### 8.5. Automotive: Drag Reduction and Structural Adaptability

In automotive design, drag-reducing morphing body panels and active cooling systems enhance fuel efficiency and safety [263]. The benefits include:

- 7–12% reduction in fuel consumption [125]
- Lower CO_2_ emissions [264]
- Improved vehicle stability and control under varying aerodynamic loads

Such features are especially promising for electric vehicles, where energy conservation is directly linked to range performance.

### 8.6. Construction: Energy-Smart Façades for Climate Adaptation

Buildings incorporating morphing architectural elements—such as bioinspired adaptive louvers or thermally responsive skins—improve environmental comfort while reducing energy loads. The benefits include:

- 15% HVAC energy reduction [265]
- 20% decrease in overall energy use [266]
- Mitigation of urban heat island effects via dynamic shading [267]

These systems incorporate natural motifs, such as the hygroscopic movements of pinecones or thermoregulatory responses of desert flora, which exhibit reactive opening or closing mechanisms relative to humidity or temperature, making them well-suited for applications in net-zero energy buildings.

### 8.7. Agriculture: Adaptive Systems for Precision Farming

Morphing agricultural structures, such as solar-tracking covers or humidity-sensitive shade systems, help optimize environmental conditions for crops. These innovations support:

- 30% reduction in irrigation energy
- 25% decrease in water use
- Improved crop yield and resilience in extreme weather

By mimicking heliotropic plant behavior, morphing systems can enable more sustainable and climate-resilient farming operations.

### 8.8. Health Care: Bioadaptive Mobility Solutions

In healthcare, adaptive prosthetics and wearable robotics incorporating morphing principles improve user comfort, gait precision, and rehabilitation outcomes. Other benefits include:

- 40% reduction in maintenance costs
- Enhanced adaptability to terrain and posture
- Enhanced quality of life for users with mobility impairments

These systems mirror muscle-tendon interactions and offer programmable stiffness, making them ideal for aging populations and neurorehabilitation applications [268,269].

### 8.9. Defense: Agile and Stealth-Optimized Systems

Stealth drones and morphing aerial vehicles developed for defense applications benefit from adaptive geometries that improve maneuverability and reduce detectability. The benefits include:

- 25% decrease in operational costs via fuel savings and maintenance [270]
- Enhanced agility in turbulent or constrained airspace [271]
- Reduced radar and acoustic signature, supporting covert operations

Such capabilities are vital for reconnaissance missions in contested or disaster-prone environments.

### 8.10. Space Exploration: Deployable and Resilient Habitats

Morphing systems in extraterrestrial applications allow compact stowage during launch and adaptive reconfiguration upon deployment. The benefits include:

- 33% reduction in launch volume [272]
- Improved radiation and debris shielding [273]
- Foundation for adaptive living modules on the Moon or Mars

Biomimetic strategies help address the challenges of reduced gravity, resource constraints, and long-duration missions in space.

### 8.11. Urban Infrastructure: Adaptive Resilience to Climate Stressors

Urban resilience is enhanced via morphing flood barriers, dynamic seawalls, and flexible transport systems designed to respond to environmental changes. Benefits include:

- 50% reduction in flood damage
- Modular designs to accommodate sea-level rise and storm surge
- Support for climate-smart cities with flexible infrastructure responses

These systems help safeguard critical infrastructure while minimizing permanent structural changes [274,275].

### 8.12. Consumer Electronics: Flexibility Meets Sustainability

In the rapidly evolving field of consumer electronics, morphing elements support innovations such as foldable displays and self-adjusting wearables, which result in:

- 20% cost reduction in flexible screen production [276]
- 15% decrease in electronic waste [277]
- Extended device lifespans via structural adaptability [278]

Nature-inspired durability and motion strategies can revolutionize how devices interact with human ergonomics and aging materials.

### 8.13. Summary and Outlook

Bioinspired morphing systems are redefining technological adaptability across transport, energy, healthcare, and robotics fields, with real-world impact spanning energy conservation, resource efficiency, disaster preparedness, and improved quality of life. Figure 23 presents a snapshot of this transformation, where design principles extracted from nature enhance function, sustainability, and resilience across diverse domains. The transition from experimental prototypes to commercial systems is accelerating with advancements in materials science, control systems, and manufacturing technologies. Morphing systems are transitioning from niche innovations to foundational technologies shaping 21st-century engineering.

## 9. Conclusions

Bio-inspired morphing offers a transformative paradigm for engineering systems operating in fluid environments. By emulating the adaptive strategies of natural flyers and swimmers, these systems dynamically alter their shape to optimize performance across diverse operating conditions.

The inherent functional principles of biological structures and their sophisticated control mechanisms, refined over millions of years, have inspired novel engineering solutions. This synergy between biology, materials science, and fluid dynamics is crucial for developing flexible, responsive systems.

Over the past decade (2015–2025), significant advancements have propelled bio-inspired morphing from concept to tangible prototypes. Innovations in smart materials (e.g., advanced multifunctional composites, electroactive polymers), high-fidelity computational modeling, and model-based adaptive control have enabled systems demonstrating notable gains, including up to a 30% increase in lift-to-drag ratio, 4 dB noise reduction, and a 15% boost in propulsive or power-capture efficiency. These developments have led to successful real-world trials in aerospace and renewable energy applications, showcasing their viability for enhanced efficiency and reduced environmental impact.

Despite these successes, challenges persist in material durability, complex actuation and control schemes, and regulatory frameworks. However, emerging technologies like self-healing polymers, 4D printing, and AI-driven optimization (e.g., machine learning and model predictive control) promise to overcome these barriers. The continued integration of partial morphing and the exploration of soft robotics further expand the potential for highly adaptable and resilient fluid systems.

Ultimately, bioinspired morphing underscores that engineering can transcend rigid design by embracing nature’s distributed control and flexibility. This interdisciplinary pursuit is poised to reshape aeronautics, hydrodynamics, and renewable energy, delivering more sustainable, versatile, and efficient solutions for decades to come.

## Figures and Tables

**Table 1:** Emerging smart materials for underwater applications.

Technology	Capabilities	Depth Rating	Source
Marine Skin v2	Depth/temperature/salinity sensing	2000 m	[97,98]
Magneto-resistive textiles	Submerged, touchless control interfaces	Washable	[99]

**Table 2:** Biological inspirations morphing concepts, engineering analogues, key metrics, innovation potential, and performance outcome (Successful/Partial).

Organism	Morphing Mechanism	Key Parameters	Engineering Analogue	Performance Gain	Innovation Potential	Outcome
Albatross	Wing extension for dynamic soaring	Aspect ratio: 18–22	Long-endurance UAV wings	30% drag reduction in crosswinds	Solar-integrated morphing skins	Successful
Dragonfly	Corrugated wing leading edge	LEV stability Re: 1.03 × 10^5^,	Micro-drone wing designs	25% lift enhancement at low Re	3D-printed corrugated nanocomposites	Successful
Humpback Whale	Tubercled flipper leading edge	Tubercle wavelength: 12% chord	Wind turbine blades	32% stall delay, 8% lift increase	Active morphing tubercles (SMA)	Successful
Dandelion Seed	Bristly pappus for passive lift	Porosity: 85%, Re: 1.02 × 10^5^	Drag-reducing aerial sensors	40% longer airborne duration	Biodegradable polymer bristles	Successful
Octopus	Mantle contraction for jet propulsion	Thrust efficiency: 68%, Re: 1.04 × 10^5^	Soft underwater thrusters	55% faster acceleration	Electroactive polymer artificial muscles	Successful
Peregrine Falcon	Alula for flow separation control	Angle of attack: 15°, Re: 1.05 × 10^5^	Aircraft leading-edge slats	20% stall speed reduction	Adaptive alula-inspired flaps	Successful
Humming bird	Wing reversal for hovering	Wingbeat frequency: 79 Hz	Flapping-wing drones	Power increases by 3.2 times	Resonant piezoelectric actuators	Successful
Boxfish	Rigid carapace with hydrodynamic shape	Drag coefficient: 0.06	Submarine hull design	18% drag reduction at Re 1.06 × 10^5^	3D-printed biomimetic hulls	Successful
Beetle	Elytra deployment for wing protection	Deployment time: 0.2 s	Retractable UAV wings	25% storage volume reduction	Shape-memory alloy hinges	Successful
Jellyfish	Bell pulsation for efficient propulsion	Propulsive efficiency: 48%	Soft robotic swimmers	30% energy savings	Dielectric elastomer actuators	Successful
Gecko	Toe adhesion via van der Waals forces	Adhesion force: 10 N/cm^2^	Climbing robots	50% surface adaptability	Microstructure polymer adhesives	Successful
Shark	Dermal denticles for drag reduction	Skin roughness: 0.1 µm	Ship hull coating	12% fuel efficiency gain	Laser-etched biomimetic surfaces	Partially Successful

**Table 3:** Aerospace morphing technologies, their bio-inspiration, actuation method, quantified performance benefit, and quick outcome verdict (Successful/Partial).

Functionality	System	Bio- Inspiration	Actuation	Performance Benefit	Case Study	Outcome
Lift Enhancement	Variable Camber Wings	Bird covert feathers	SMA ribs	Camber ±8°, ↑18% L/D ratio	NASA MAW	Successful
	Telescopic Wings	Swift retraction	Hydraulic rods	+40% span, 15% fuel saving	Airbus Albatross	Successful
Drag Reduction	Adaptive Winglets	Eagle feathers	Piezo actuators	Twist ±15°, ↓12% induced drag	Boeing Eco Demonstrator	Successful
	Variable-Sweep Wings	Swift wing dynamics	Electromagnetic	Sweep 20–60°, ↓18% drag	F-14 Retrofit	Successful
	Morphing Skins	Fish scales	Elastomer-composite	Smooth airflow, durable under strain	Lockheed Martin Morphing Skin	Successful
	Vortex Generators	Shark denticles	MEMS	↑10% boundary layer stability	NASA Flow Control	Partially Successful
Noise Suppression	Leading Edge Serrations	Owl feathers	Compliant 3D-printed	↓10 dB noise at 1 kHz	Airbus Silent Falcon	Successful
Control and Maneuverability	Inflatable Wingtips	Bat wings	Pneumatic muscles	↑22% roll authority	DARPA Flex Foil	Successful
	Deployable Airbrakes	Peacock tail	Shape memory polymers	90° deploy, ↑30% braking efficiency	BAE Adaptive Airbrake	Successful
	Flapping Wing UAVs	Insect thoracic muscles	Piezo flaps	120 Hz flapping, ↑25% thrust	Harvard RoboBee	Successful
Energy Efficiency	Active Twist Rotors	Dragonfly wings	Piezo fiber composites	±10° twist, ↓20% vibration	Sikorsky Active Rotor	Partially Successful
	Morphing Engine Inlets	Whale baleen	Adaptive polymer louvers	±25% flow, ↑15% compressor efficiency	GE Adaptive Jet Engine	Successful

Symbol Key: ↑ increase, ↓ decrease, + additive change, ± plus-or-minus variation relative to the baseline.

**Table 4:** Key actuation characteristics, performance metrics, cost, and morphing-use suitability of Shape Memory Alloys (SMAs), Piezoelectric Materials (PZTs), and Electroactive Polymers (EAPs) for bioinspired structures.

Property	SMA (Shape Memory Alloy)	PZT (Piezoelectric)	EAP (Electroactive Polymer)
Actuation Method	Thermal activation (phase transition)	Electric field (inverse piezo effect)	Electric field (ionic/electrostatic)
Strain Capability	High (~4–8%)	Very low (~0.1%)	High (~10–30%)
Force Output	High	Very high	Low to moderate
Response Speed	Slow	Fast	Moderate
Control Precision	Low	High	Moderate
Fatigue Durability	Moderate	High	Low–moderate
Cost	Moderate	High	Low–moderate
Typical Use in Morphing	Large-scale, reversible shape changes (e.g., morphing wings or skins)	High-frequency flapping or surface vibration	Soft actuators for flexible fins, skins, or artificial muscles

**Table 5:** Integrated Research-Methodology Matrix for Bioinspired Morphing Systems.

Tier	Method/Tool	Key Capability	Typical Use-Case	Reference
Theoretical	Potential-flow + small-deflection beam theory	Rapid analytic lift-pressure and bending-stress estimates for minor camber/twist changes	First-pass screening of variable-camber wing sections	[181]
	Lighthill’s elongated-body theory	Closed form thrust and efficiency of undulatory bodies	Conceptual sizing of fish-like propulsors	[232]
	Multi-scale homogenization of smart-material lattices	Links actuator micro-physics to global stiffness, mass, and actuation limits	SMA-lattice camber-morphing wings	[233]
Computational	Dynamic-meshing RANS/LES CFD	Resolves time-accurate vortex dynamics around moving geometry	Optimizing camber-schedule at Re ≈ 10^6^	[234]
	Partitioned FSI (solids4Foam + OpenFOAM)	Two-way coupling of fluid loads and large structural deflection	Flexible fin or wingtip bending simulations	[235]
	Vortex-lattice/discrete-vortex methods	Fast unsteady force prediction with negligible meshing cost	Mission-level optimization for UAV morphing plans	[236]
Experimental	Wind-tunnel PIV on flexible/membrane wings	Full-field velocity and pressure validation of CFD/FSI models	Membrane-wing micro-air-vehicle prototypes	[237]
	Free-swimming soft-robotic fish in a water flume	Net thrust, efficiency, and kinematics of compliant fins	Dolphin-fluke or caudal-fin morphing tests	[238]
	SMA coupon/sub-component tests	Actuation strain, hysteresis, and fatigue life of smart skins	Shape-memory alloy spar for adaptive trailing-edge	[239]
AI and ML	Surrogate-assisted genetic algorithm (Kriging + NSGA-II)	Pareto front discovery with 100× fewer CFD calls	Multi-objective wing-shape and sweep optimization	[223]
	Deep reinforcement learning (DQN/PPO)	Real-time gust rejection via autonomous camber change	Morphing rotor blades/UAV gust alleviation	[225]
	Physics-informed CNN (flow ROM)	Instantaneous flow-field reconstruction from sparse sensors	Closed-loop stall detection and control	[240]

**Table 6:** Durability Challenges and Mitigation Strategies.

Key Failure Modes	Mitigation Strategies	Reference
Microcracks from cyclic bending	Fatigue-resistant alloys; CNT-reinforced shape-memory polymers	[241]
Composite delamination under peel/shear	Reinforced resin matrices; z-pin or stitching reinforcement	[242]
Adhesive creep and hinge wear	Self-lubricating polymers; sealed hinge housings	[134]
UV-induced polymer embrittlement	UV-stabilizing coatings; carbon-black-loaded skins	[243]

**Table 7:** Flexibility vs. Strength Trade-Offs and Design Solutions.

Design Dilemma	Short-Term Solutions
Overly compliant skins deform uncontrollably	Hybrid multi-material laminates; local stiffening ribs
Excessively stiff skin requires a high actuator force	Variable-stiffness layers, graded fiber orientations
Tools	Where to Apply
FEA to map stress/strain	Identify “soft zones” vs. load paths
Topology optimization	Discard unnecessary material in low-stress regions

**Table 8:** Environmental Stressors and Protective Strategies.

Environmental Stressor	Protective Strategy	Reference
UV embrittlement of polymers	UV-absorbing coatings; carbon-black additives	[244]
Saltwater corrosion of actuators	Sacrificial anti-corrosion layers; sealed housings	[245]
Thermal cycling mismatch	Thermal-expansion-matched hybrid materials	[246]
Chemical attack on adhesives	Chemically resistant polymers; self-healing resins	[247]

**Table 9:** Manufacturing and Scalability Challenges and Short-Term Mitigations for Bioinspired Morphing Systems.

Challenge	Short-Term Mitigation	Reference
Complex multi-material assembly	Automated fiber placement; 3D multi-material printing; inline metrology	[248]
Embedding sensors and electronics	Direct-write printed electronics; sealed cable tracks	[249]
Complex multi-material assembly	Automated fiber placement; 3D multi-material printing; in-line metrology	[196]
Certification and regulatory overhead	Pilot industry–regulator collaborations; shared testbeds	[250]
Workforce skill gaps for novel processes	AR/VR-based operator training; digital apprenticeships	[251]
Supply-chain variability for smart materials	Multi-source qualification; buffer inventories	[252]

**Table 10:** FSI Complexities and Modeling/Control Solutions.

FSI Complexity	Modeling/Control Strategy
Vortex shedding ↔ structural deformation	High-fidelity CFD/FEA co-simulation; reduced-order FSI
Nonlinear, multi-scale time dynamics	Partitioned solvers; phase-lagged ROMs
Risk of aero-elastic instabilities	Real-time stability monitoring; passive adaptivity

**Table 11:** Actuation Trade-offs and Energy-Saving Strategies.

Actuation Trade-Off	Energy-Saving Strategy
High-power hydraulics/SMAs	Bi-stable snap-through; elastic strain energy storage
Slow SMA response vs. fast piezo	Multi-actuator hybrids; passive morphing alignment
Continuous power for hold	Latching mechanisms; bistable structures

**Table 12:** Real-Time Control Challenges and Advanced Solutions.

Control Challenge	Advanced Solution
Highly nonlinear, over-actuated morphing systems	INDI with QP allocation; real-time MPC
Sensor noise, latency, model uncertainty	Kalman/PINN-based observers; domain randomization
Actuator bandwidth limits	Predictive control; sensor-actuator co-design

**Table 13:** Challenges and Mitigation Strategies for Bioinspired Morphing Systems.

Challenge	Biological Insight	Short-Term Solution	Long-Term Vision	Key Innovation Needed
Fatigue in flexible skins	Collagen-elastin networks in bats	Carbon nanotube-reinforced SMPs	Self-healing vascularized materials	3D-bioprinted hybrid composites
Energy-intensive actuation	Resonant insect flight muscles	Regenerative piezoelectric systems	Biohybrid muscles (cells + polymers)	Mitochondrial-inspired energy storage
Real-time FSI control	Fish lateral line sensing	MEMS pressure sensor arrays	Neuromorphic computing chips	Quantum-inspired flow prediction
Scalability to large systems	Whale fluke biomechanics	Modular morphing subcomponents	Distributed nanoactuator networks	Metamaterial-based structural logic
Environmental degradation	Self-cleaning lotus leaves	Superhydrophobic coatings	Photocatalytic self-cleaning skins	TiO_2_ nanoparticle integration
High manufacturing costs	Termite mound construction	3D-printed hierarchical structures	Self-assembling materials	DNA-origami-inspired fabrication
Sensor integration complexity	Spider sensory hairs	Optical fiber Bragg gratings	Distributed neural-like sensor nets	Bioinspired optoelectronic skins
Limited morphing range	Chameleon skin chromatophores	Multilayer dielectric elastomers	Programmable color/shape morphing	Quantum dot-embedded polymers
Thermal management	Elephant ear convection	Phase-change material cooling	Microfluidic cooling channels	Biomimetic vascular networks
Noise generation	Owl feather serrations	Micro-perforated trailing edges	Active noise-cancelling surfaces	Meta-material acoustic cloaking
Regulatory compliance	Bird migratory patterns	Adaptive certification frameworks	Global morphing standards	AI-driven regulatory sandboxes
Public acceptance	Butterfly aesthetic patterns	Art-integrated morphing designs	Emotion-responsive aesthetics	Neuroaesthetic design principles

## Data Availability

No new data were created.
